# Colorectal cancer cell intrinsic fibroblast activation protein alpha binds to Enolase1 and activates NF-κB pathway to promote metastasis

**DOI:** 10.1038/s41419-021-03823-4

**Published:** 2021-05-25

**Authors:** Ziming Yuan, Hanqing Hu, Yihao Zhu, Weiyuan Zhang, Qingxiao Fang, Tianyu Qiao, Tianyi Ma, Meng Wang, Rui Huang, Qingchao Tang, Feng Gao, Chaoxia Zou, Xu Gao, Guiyu Wang, Xishan Wang

**Affiliations:** 1 Colorectal Cancer Surgery Department, The Second Affiliated Hospital of Harbin Medical University, Harbin, Heilongjiang China; 2 Department of Biochemistry and Molecular Biology, Harbin Medical University, Harbin, Heilongjiang China; 3 Department of Colorectal Surgery, National Cancer Center/National Clinical Research Center for Cancer/Cancer Hospital, Chinese Academy of Medical Sciences and Peking Union Medical College, Beijing, China

**Keywords:** Cancer immunotherapy, Non-coding RNAs

## Abstract

Fibroblast activation protein alpha (FAP) is a marker of cancer-associated fibroblast, which is also expressed in cancer epithelial cells. However, the role of FAP in colorectal cancer (CRC) cells remains to be elucidated. Here we investigate the expression pattern of FAP in CRC tissues and cells to prove that FAP is upregulated in CRC cells. Loss- of and gain-of-function assays identified FAP promotes migration and invasion instead of an effect on cell proliferation. Microarray assays are adopted to identify the different expressed genes after FAP knockdown and gene set enrichment analysis (GSEA) is used to exploit the involved signaling pathway. Our works reveal FAP exerts a function dependent on NF-κB signaling pathway and FAP expression is associated with NF-κB signaling pathway in clinical samples. Our work shows FAP is secreted by CRC cells and soluble FAP could promote metastasis. To investigate the mechanism of FAP influencing the NF-κB signaling pathway, LC/MS is performed to identify the proteins interacting with FAP. We find that FAP binds to ENO1 and activates NF-κB signaling pathway dependent on ENO1. Blocking ENO1 could partially reverse the pro-metastatic effect mediated by FAP. We also provide evidences that both FAP and ENO1 are associated with CRC stages, and high levels of FAP and ENO1 predict a poor survival in CRC patients. In summary, our work could provide a novel mechanism of FAP in CRC cells and a potential strategy for treatment of metastatic CRC.

## Introduction

Colorectal cancer (CRC) has become one of the most common malignancies with being the third leading cause of cancer-related death worldwide and is characterized by poor prognosis and treatment^1^. Although there has been considerable advancement in the management of CRC, treatment options have limited impact on cure rates and long-term survival. Metastasis is one of the main reasons of death in CRC patients. The liver is the most common organ for CRC metastasis and patients with liver metastasis have much poorer prognosis. Therefore, lucubrating the mechanism of liver metastasis is crucial.

Abundant evidences have indicated that tumor microenvironment (TME) plays a crucial role in cancer progression^2^. The co-evolution of cancer cells and stromal functional cells or molecules constitutes significant hallmarks of cancer^3^. Liver metastasis is also a complicated process involving the interaction between stromal cells and cancer cells. Previous research has shown cancer-associated fibroblast (CAF), a main member of cancer stroma, acted as a key character in TME and promoted the metastasis in many kinds of tumor. CAF-derived WNT2 increases tumor angiogenesis in colon cancer^4^. Pelon et al.^5^ reported that CAF heterogeneity in axillary lymph nodes drives metastases in breast cancer.

Fibroblast activation protein-α (FAP) is the hallmark of CAF including CRC CAF. It is a homodimeric integral membrane gelatinase of the serine protease family and is selectively expressed by CAFs in stromal compartment^6^. In addition to a stromal cell marker, FAP has been found in multiple epithelial cancer cell lines^7–9^. Moreover, FAP also highly expressed in cancer cells and play an important role in regulating cancer cell biology. Stromal FAP promotes gastric cancer progression via epithelial–mesenchymal transition through Wnt/β-catenin pathway^10^. FAP is more abundant in malignant neoplasms than in benign ovarian neoplasms, as well as in moderately differentiated and undifferentiated ovarian carcinomas compared to well-differentiated neoplasms^11^. Increased FAP expression was found in a subgroup of high-grade gliomas and was most elevated in the mesenchymal subtype of glioblastoma with poor survival^9^. However, the role of FAP in CRC, especially in metastasis CRC, remained to be elucidated.

Our works confirmed FAP was highly expressed in CRC cells and found that FAP interacted with ENO1 to promote the CRC migration and invasion via nuclear factor-κB (NF-κB) pathway.

## Methods

### Cell culture and primary cell isolation

Human CRC cell lines were all purchased from Chinese Academy of Science’ cell bank (Shanghai, China). HCT116 was cultured in McCoy’s 5A medium (Jiangsu Kaiji Biotechnology Co., Jiangsu, China) and the other cell lines were grown in Dulbecco’s modified Eagle’s medium (DMEM, Gibco Laboratories, Grand Island, NY). All cell mediums add 10% fetal bovine serum (FBS, GIBCO, Carlsbad, CA) and cells were cultured at 37 °C in a humidified incubator with 5% CO_2_.

Primary cancer epithelial cells and CAFs were obtained from CRC samples and adjacent normal tissues by radical surgery. Briefly, the tumor and normal samples were washed three times with phosphate-buffered saline (PBS), minced, and digested by collagenase type I, collagenase II, and hyaluronidase (1.5 mg/ml, Sigma-Aldrich) at 37 °C with gentle agitation for 3 h in DMEM medium with 10% FBS. To get pure fibroblasts, magnetic-activated cell sorting (MACS) with anti-fibroblast-specific protein microbeads were used to isolate pure fibroblast. The number of cells was counted and 107 cells were incubated with 20 μl microbeads, mixed well, and incubated for 30 min at room temperature. Cells were washed with 1 ml PBS buffer and centrifuged at 300 × *g* for 10 min. Aspirate supernatant was completely resuspended in 3 ml PBS. The solution flowed through the LS column according to the manufacturer’s protocols. On the other hand, the cancer cells and normal epithelial cells were isolated with anti-CD326 microbeads.

The membrane protein and cytoplasmic protein were isolated by Mem-PER (Thermo Fisher Scientific, Waltham, MA) according to the standard protocol of the manufacturer. Briefly, the indicated number of cells were suspended in Cell Wash Solution buffer and centrifuged, carefully removed, and the supernatant discarded. Next, Permeabilization Buffer was added to the cell pellet, vortexed, and incubated for 10 min at 4 °C, to obtain a homogeneous cell suspension. The suspension was centrifuged to acquire cytosolic proteins and Solubilization Buffer was added to the pellet. The pellet was vortexed and incubated at 4 °C for 30 min, then centrifuged to acquire the supernatant as membrane proteins.

### RNA extraction and quantitative reverse-transcriptase PCR analysis

Total RNA was extracted from CRC cell lines with TRIzol reagent (Invitrogen, Carlsbad, CA, USA) and 2 μg total RNA was reverse-transcribed using the High Capacity cDNA Reverse Transcription Kit (Applied Biosystems, Foster City, CA) according to the manufacturer’s instructions. DNA was quantified using Nanodrop 2000 spectrophotometer (Thermo Fisher Scientific, Waltham, MA). Quantitative reverse-transcriptase PCR was performed with a reaction mix of SYBR Green (Thermo Fisher Scientific, Waltham, MA) in triplicate using the Applied Biosystem 7500 quantitative PCR (qPCR) system (Applied Biosystems). Relative expression was compared by ΔCt method and β-actin served as the endogenous gene. The sequences of primer in this study are shown in Supplemental Table 1.

### Cell transfection

Lenti-shFAP, lenti-FAP, and their corresponding control vectors were designed and purchased from GeneChem (Shanghai, China). Following transfection, transfected successfully cells were selected in the presence of puromycin (Sigma-Aldrich Corp., St. Louis, MO, USA) for 2 weeks. The sequences of short hairpin RNAs are shown in Supplemental Table 1.

For small interfering RNAs (siRNAs) or plasmid transfection, Lipofectamine 2000 reagent was used according to the manufacturer’s protocol and the sequences of siRNAs are shown in Supplemental Table 1.

The mutated p65 was synthetized by Genechem Corporation (Shanghai, China). The sequence of mutated p65 was shown in Supplemental File 1.

### Western blotting

Western blotting was performed using standard techniques as described previously^12^. Total cell extracts were collected and quantified using BCA Protein Assay Kit (Wanlei Bio, Shenyang, China) according to the manufacturer’s protocols. Fifty micrograms of proteins were electrophoresed through 10% SDS polyacrylamide gels and then transferred onto polyvinylidene difluoride membranes (Millipore, USA). The membranes were blocked with 5% non-fat milk in Tris-buffered saline with Tween 20 for 2 h at room temperature and incubated with primary antibodies overnight at 4 °C. Secondary antibodies labeled with horseradish peroxidase (HRP) were used to incubate the membrane at room temperature for 2 h and the signals were detected using ECL Kit (Wanlei Bio, Shenyang, China). Subsequently, the images were analyzed by Bio-Rad ChemiDoc MP system. ACTIN antibody was used as internal standard for the whole-cell lysates. The details of antibodies were listed in Supplemental Table 2. The band intensity was analyzed by ImageJ software and the relative expression was defined as the comparison of experiment group to control group. All the western blotting assays were performed by three independent replicates and an average of three independent assays were used for statistical analyses.

### Immunohistochemistry

Paraffin-embedded samples were prepared at 4 μm thickness. All the slides were through deparaffinization and rehydration. Antigen retrieval was performed by a pressure cooker for 20 min in 0.01 M citrate buffer (pH 6) to remover aldehyde links. The slides were incubated with ENO1 antibody (1 : 50) overnight. HRP-labeled secondary antibody was incubated for 1 h and immunodetection was performed using diaminobenzidine on the next day according to the manufacturer’s protocol. The immunohistochemistry score (SI) was calculated by the staining intensity (0, negative; 1, weak; 2, moderate; 3, strong) multiplied by the positive rate of stained cells (0–5%, 0; 6–25%, 1; 26–50%, 2; 51–75%, 3; >75%, 4). In this study, SI = 4–12 was defined as positive staining, whereas SI = 0–3 was defined as negative staining.

### Immunofluorescence analysis

The cells were seeded on clear coverslips, fixed with 4% paraformaldehyde for 30 min at room temperature, followed by permeabilization and blockade with 1% bovine serum albumin plus 0.1% Triton X-100. The cells were incubated with anti-FAP antibodies (Abcam) at 4 °C overnight. Then, the coverslips were incubated with fluorescent secondary antibodies (Invitrogen) for 1 h at room temperature in the dark room. Ultimately, cell nuclei were counterstained with 4,6-diamidino-2-phenylindole and the stained sections were photographed by a microscope (FSX100, Olympus).

### Cell migration and invasion assays

The cell migration assay was performed using the Transwell Chamber from BD Biosciences (SanJose, CA, USA). Briefly, cells (0.5~2 × 10^5^) were seeded in the upper chamber in serum-free media. The cell invasion assay was performed nearly the same as the migration assay. Cells number is 0.5~2 × 10^6^. Matrigel (Corning, USA) was tiled in the upper chamber before the seeded cells. The lower chamber was filled with corresponding media with 10% FBS as a chemoattractant. After 24~48 h, cells that invaded through the membrane were fixed with 4% paraformaldehyde for 10 min at room temperature, and then stained with 0.5% crystal violet and counted by ImageJ software according to a previous report^13^.

### Whole human genome expression microarray analysis

Total RNA of SW480 cells were extracted and purified by RNeasy Mini kit (Qiagen) following the manufacturer’s instruction. The preparation of samples and hybridization were conducted according to standard protocol^14^. One microgram of RNA was amplified, transcribed, and labeled with fluorescence following the manufacturer’s Agilent’s Quick Amp Labeling protocol, version 5.7. After the slides were washed, the arrays were scanned with the Agilent Scanner G2505C. The different expression genes were analyzed using Agilent Whole Genome Microarray (4 × 4.4k, version 2.0) by Agilent Genespring GX v2.1 software. The threshold we used to identify upregulated or downregulated mRNAs is fold change > 2 or fold change < 0.5.

### Gene set enrichment analysis

Gene set enrichment analysis was performed to reveal the molecular pathway associated with FAP knockdown in SW480 cells using GESA software (version 4.0.3) according to the Oncogenic Signatures gene set (c6.all.v7.2.symbols.gmt).

### Co-immunoprecipitation and liquid chromatography mass spectrometry

Total proteins were extracted with cell lysis buffer (Cell Signaling Technology, Danvers, MA) supplemented with protease inhibitor and phosphatase inhibitor (Cell Signaling Technology, Danvers, MA). Lysate was incubated with anti-FAP (R&D Systems, USA), anti-ENO1, and IgG antibodies (as a negative control) at 4 °C overnight, with gentle rotation. Then, the protein–antibody complexes were incubated with protein A agarose (Cell Signaling Technology, Danvers, MA) for 5 h at 4 °C with gentle rotation. Immunoprecipitates were then collected by centrifugation at 14000 × *g* for 30 s at 4 °C, after which the bead complexes were washed three times with cell lysis buffer. After the final wash, protein A agarose were eluted by boiling in 5× SDS sample buffer at 100 °C for 5 min before western blot analysis. The immunoprecipitates were prepared according to a previous study^15^ and the tryptic peptides were analyzed by Orbitrap Fusion LUMOS mass spectrometer (Thermo Fisher Scientific). All spectra were analyzed using PEAKS 8.0 (Bioinformatics Solutions), for processing, de novo sequencing and database searching. The resulting sequences were searched against the UniProt Human Proteome database. The data of liquid chromatography mass spectrometry (LC/MS) were listed in Supplemental Table 3.

### Enzyme-linked immunosorbent assay

Cells were cultured in complete medium with 10% FBS till 80% confluency. The medium was discarded and the cells were washed by PBS three times and cultured in the serum-free medium. After 48 h, the medium was harvested as conditioned medium (CM) for enzyme-linked immunosorbent assay (ELISA). FAP concentration was detected using FAP DuoSet ELISA kit (R&D Systems, DY3715) according to the manufacturer’s protocol.

### In vivo liver metastasis xenograft experiments

The experiment protocol was reviewed and approved by the Committee on the Use of Live Animals of Harbin Medical University, Harbin, China. Male BALB/c nude mice (4–6 weeks) were housed five mice per cage in a pathogen-free room with a 12 h light/dark schedule at 25 °C ± 1 °C and fed chow diet and water. Ten mice were used per experimental group.

We performed liver metastasis assays in mice as previously described^16^. SW480-shNC and SW480-shFAP cells were washed with complete medium once and resuspended in PBS. The nude mice were anesthetized with pentobarbital sodium, a tiny left abdominal incision was made, and 5 × 10^6^ cells in 100 μL PBS were injected into the spleen parenchyma. ^18^F-FDG (150 μCi/100 μL) were injected into the tail veins and the liver metastatic tumors evaluated through positron emission tomography–computed tomography (PET/CT).

### Statistical analyses

The data are expressed as the mean ± SD. Statistical analyses were performed using the Student’s *t*-test or one-way analysis of variance for independent groups with Graphpad 8.0 software (GraphPad, LaJolla, CA, USA). Three independent assays were performed for each experiment in this work. The association between FAP expression and other genes was analyzed by the Spearman’s correlation coefficient. *P*-values < 0.05 were considered statistically significant.

## Results

### FAP is upregulated in CRC cells and associated with poor clinical outcomes

**Fig. 1:**
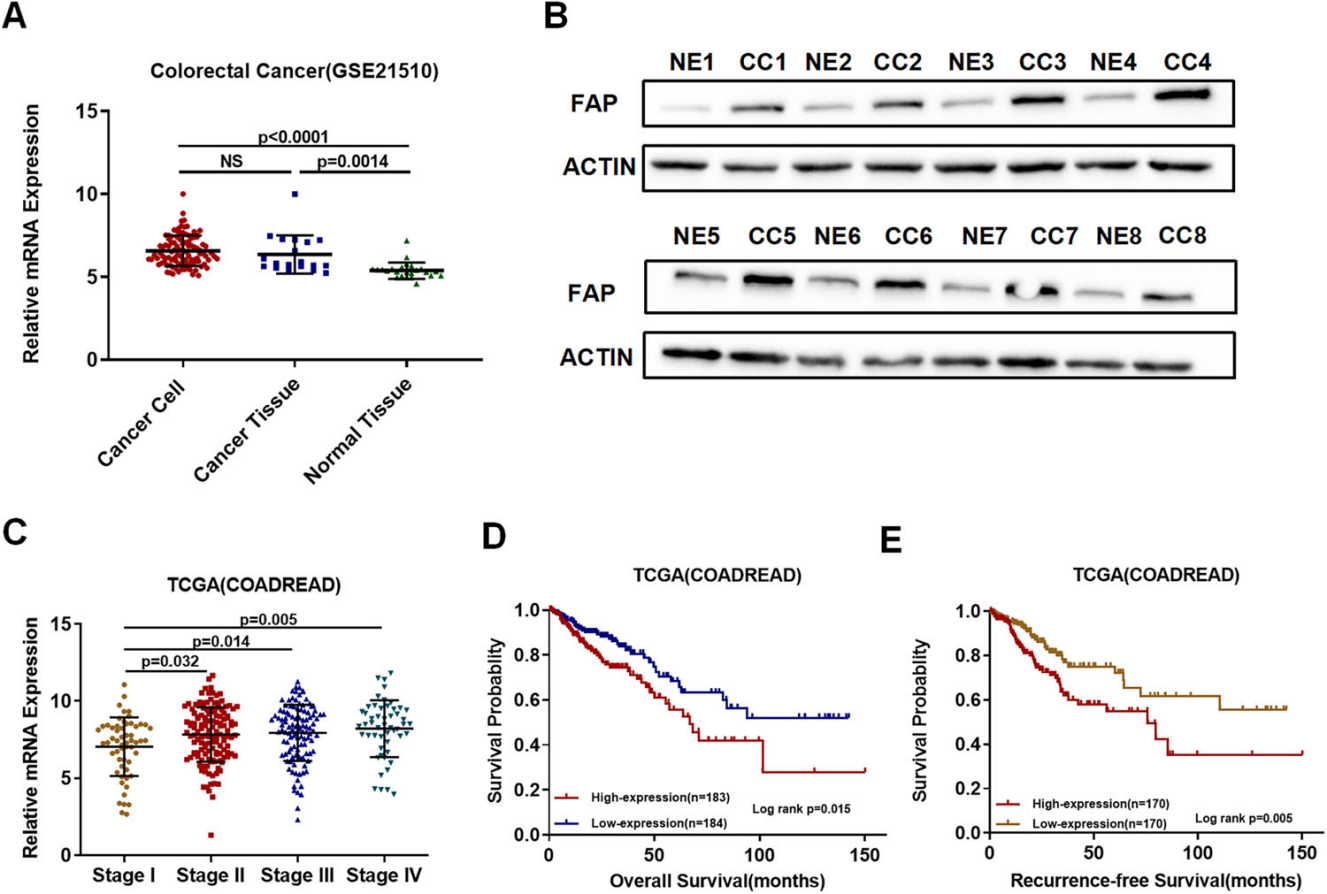
FAP is upregulated in CRC cells and associated with poor clinical outcomes. **A** Expression pattern of FAP in CRC cancer cells, cancer tissues, and normal tissues. The mRNA expression was acquired from data set (GSE21510) in Gene Expression Omnibus. Cancer cell: cells isolated from cancer tissue by LCM method. Cancer tissue: the homogenized bulk cancer tissue. Normal tissue: the homogenized bulk normal tissue. **B** Western blot analysis of FAP in cancer cells and normal colon epithelial cells isolated by MACS. CC: cancer cell; NE: normal epithelia. **C** Analysis of FAP mRNA levels in different stages of CRC patients from TCGA. **D** Overall survival of patients in FAP high-expression group and FAP low-expression group in TCGA. **E** Recurrence-free survival of patients in FAP high-expression group and FAP low-expression group in TCGA. Median FPKM was used to stratify high- and low-expression group to analyze OS and RFS.

FAP has been reported to be upregulated in many types of cancer^17–19^. From the Cancer Genome Atlas (TCGA), we found FAP was also upregulated in CRC tissues (Fig. 1A and Supplement Fig. 1A, B). In data set GSE21510 of Gene Expression Omnibus (GEO) database, cancer cells were isolated by laser-captured microdissection. FAP was highly expressed in cancer cells compared to normal tissues (Fig. 1A). We isolated normal colon epithelial cells, cancer epithelial cells, and CAFs from patient by MACS. FAP was found to be highly expressed in cancer cells compared to normal epithelial cells (Fig. 1B). In ovarian cancer, FAP was also highly expressed in cancer cells compared to normal epithelial cells (Supplemental Fig. 1C). We also found FAP expression was similar in CRC cells and CAFs (Supplemental Fig. 1D). To investigate the relationship between FAP expression and clinical characteristics, we found that FAP was increasing with the development of tumor stage (Fig. 1C). In addition, we found that the expression of FAP was associated with a shorter survival (Fig. 1D, E). Together, our data showed that FAP was highly expressed in CRC cells and associated with poor outcomes in CRC patients.

### FAP promotes CRC cell migration and invasion in vitro

**Fig. 2:**
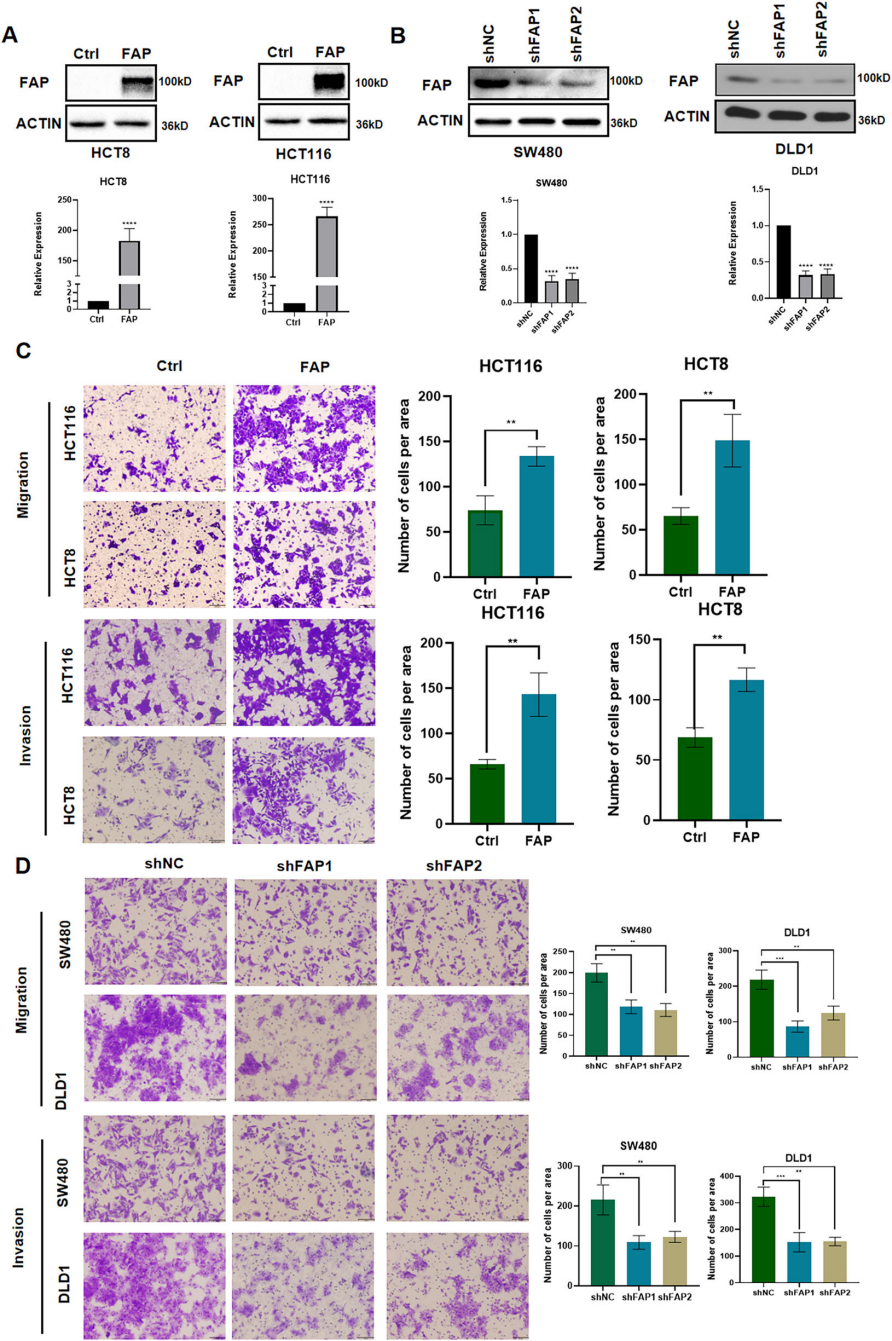
FAP promotes CRC cell migration and invasion in vitro. **A** Western blot analyzes the efficiency of FAP overexpression in HCT8 and HCT116 cells. ****p < 0.0001. **B** Western blot analyzes the efficiency of FAP knockdown in SW480 and DLD1 cells. ****p < 0.0001. **C** Migration and invasion assays of indicated cells. Error bar = 100 μm. *p < 0.05, **p < 0.01. The error bar represented the SEM. **D**. Migration and invasion assays of indicated cells. Error bar = 100 μm. **p < 0.01, ***p < 0.001. The error bar represented the SEM. Ctrl: emptor vector; FAP: FAP overexpression; shNC: negative control shRNA; shFAP: FAP knockdown shRNA.

To explore the role of FAP in CRC cell, the expression pattern of FAP was evaluated in multiple CRC cell lines (Supplemental Fig. 2A). We overexpressed FAP in HCT8 and HCT116 cell lines and knocked FAP down in SW480 and DLD1 cell lines. The efficiency was confirmed by western blotting and PCR assay (Fig. 2A, B and Supplemental Fig. 2B). Upregulation of FAP could enhance the migration and invasion of HCT8 and HCT116 cells (Fig. 2C), and downregulation of FAP could attenuate the migration and invasion of SW480 and DLD1 (Fig. 2D). The effect of FAP on cell proliferation was also evaluated. The colony formation and Cell Counting Kit-8 assays showed FAP had no influences on the proliferation (Supplemental Fig. 2C, D). Our works showed FAP promoted the migration and invasion of CRC cells in vitro.

### FAP promotes CRC liver metastasis in vivo

**Fig. 3:**
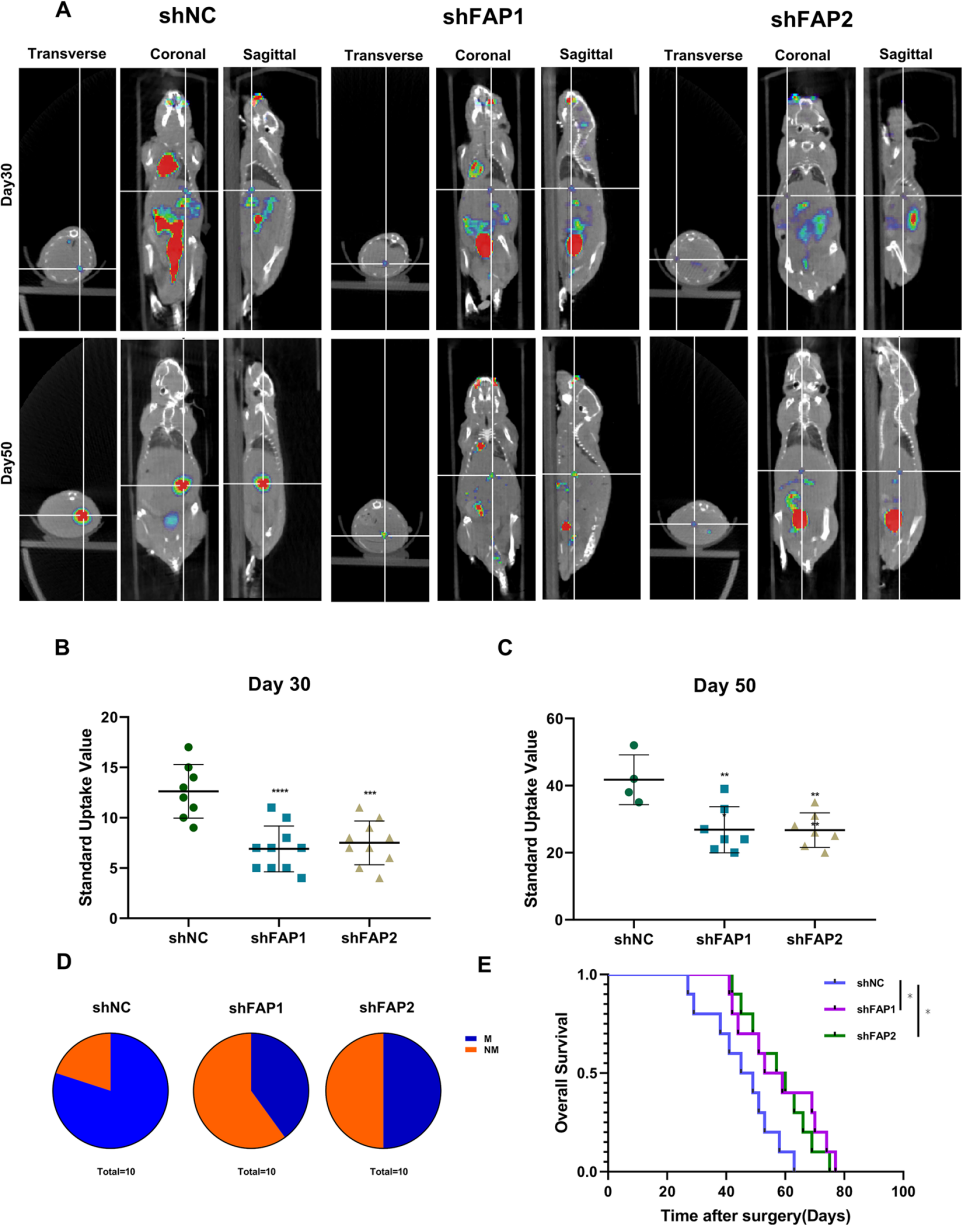
FAP promotes CRC cell migration and invasion in vivo. **A** Representative images of liver metastasis by PET/CT scan at different times. **B** Analysis of standard uptake value at 30 days after tumor cells were injected into mice spleen. ***p < 0.001, ****p < 0.0001. **C** Analysis of standard uptake value at 50 days after tumor cells were injected into mice spleen. **p < 0.001. **D** Incidence of liver metastasis formation in indicated group. NM: non-metastasis; M: metastasis. **E** Survival analysis of mice in indicated groups.

To investigate whether FAP could promote migration and invasion in vivo, we established mouse liver metastasis model by injecting the CRC cells into the spleens of Balb/c nude mice. PET/CT scan showed that knockdown of FAP retarded the metastatic formation of SW480 cells (Fig. 3A–C) and decreased incidence of liver metastasis in mice (Fig. 3C). Survival analysis also presented knockdown of FAP improved the survival (Fig. 3D). To examine the relationship of FAP with metastasis in patients, we analyzed a series of data from GEO. In data set GSE161097 and GSE51244, primary tumor and corresponding peritoneal metastatic tumor were detected by microarray. FAP was upregulated in peritoneal metastatic tumor (Supplemental Fig. 3). Our data showed that FAP promote metastasis in vivo.

### FAP exerted its function dependent on NF-κB pathway

**Fig. 4:**
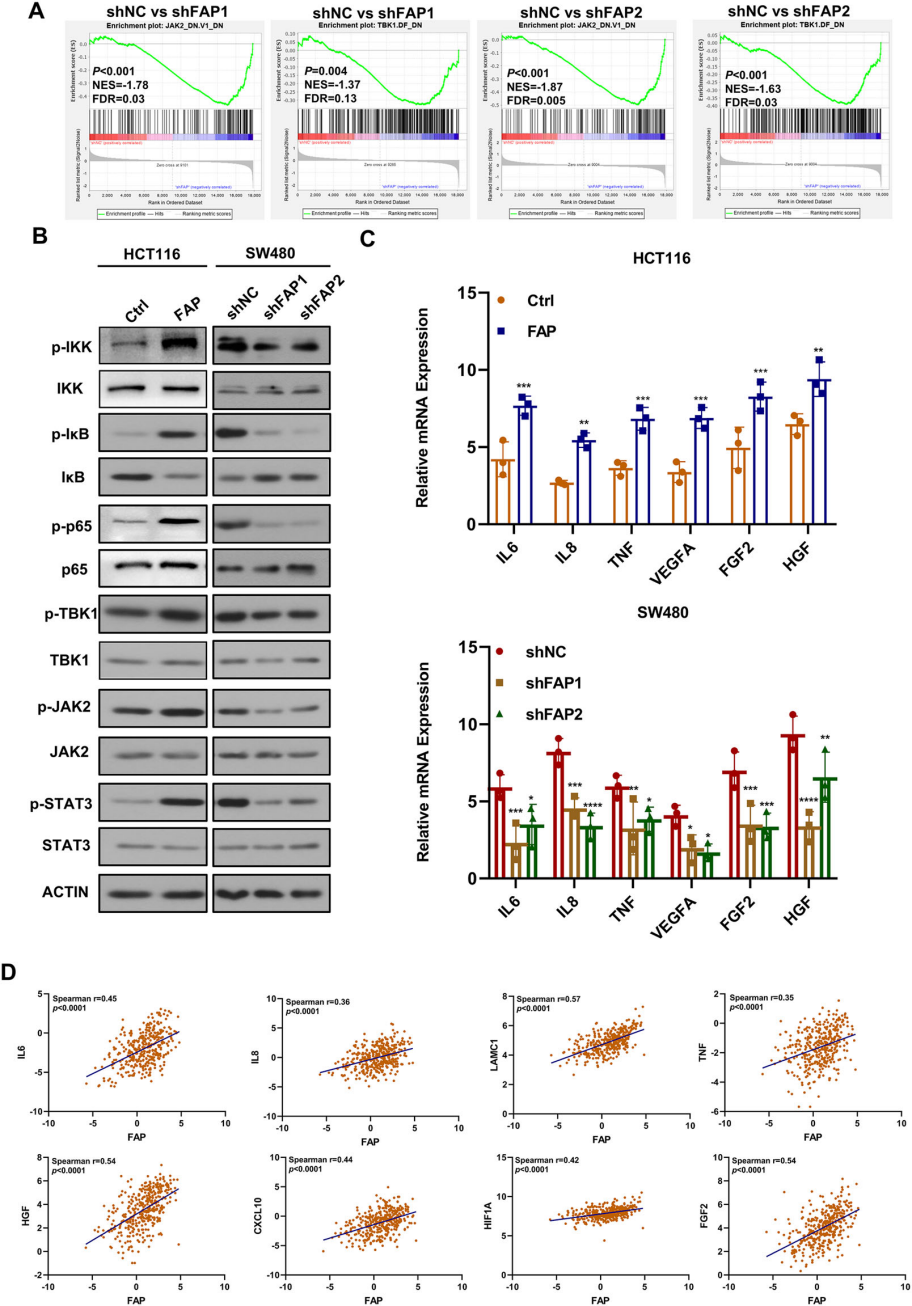
FAP exerted its function dependent on NF-κB pathway. **A** Gene set enrichment analysis in SW480-shNC, SW480-shFAP1, and SW480-shFAP2 cells using Oncogenic Signatures Gene Set (c6.all.v6.0.symbol.gmt). One thousand permutations were performed and *p* < 0.01 was considered significantly enriched. **B** Western blot analysis of the proteins in NF-κB and STAT3 pathway in indicated groups. **C** qPCR assay detected mRNA of NF-κB and STAT3 pathway-related genes in SW480 and HCT116 cells. **D** Spearman’s correlation between FAP and NF-κB, and STAT3 pathway-related genes in TCGA.

To identify the gene expression pattern associated with the FAP knockdown, we performed Agilent Whole Human Genome Array in SW480-shNC, SW480-shFAP1, and SW480-shFAP2 cells. The volcano plot showed 910 and 1233 different expression genes in shFAP1 and shFAP2 group, respectively (Supplemental Fig. 4A). Gene Ontology enrichment analysis was used to predict the different expressed genes involved in signal transduction activity and molecular transduction activity (Supplemental Fig. 4B). Gene set enrichment analysis revealed the significant enrichment of JAK2/STAT3 and TBK1/NF-κB signaling pathway (Fig. 4A). JAK2 signaling pathway has an association with TBK1/NF-κB signaling pathway. The genes that are regulated by NF-κB signaling pathway, such as *interleukin-6*, activate the JAK2/STAT3 signaling^20,21^. We speculated that FAP had an effect on NF-κB pathway. Overexpression of FAP stimulated the phosphorylation of proteins in the two pathways; however, knockdown of FAP inhibited the phosphorylation of these molecules (Fig. 4B and Supplemental Fig. 4C). qPCR assay showed FAP had an effect on the expression of genes regulated by NF-κB and JAK2/STAT3 signaling (Fig. 4C and Supplemental Fig. 4D). In the TCGA database, expression of FAP was positively correlated with genes in NF-κB and STAT3 signaling (Fig. 4D). These results showed FAP regulated the NF-κB and JAK2/STAT3 signaling cascade.

**Fig. 5:**
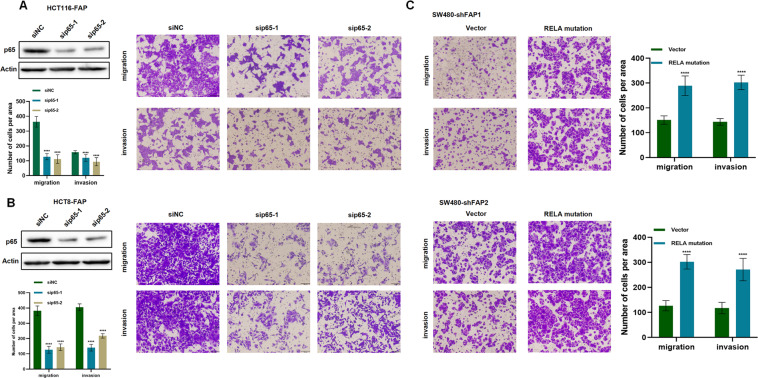
FAP exerted its function dependent on NF-κB pathway. **A** Western blot analysis of p65 knockdown in HCT116-FAP cells (left panel). Migration and invasion assays of indicated cells. Error bar = 100 μm. ****p < 0.0001 (right panel). **B** Western blot analysis of p65 knockdown in HCT8-FAP cells (left panel). Migration and invasion assays of indicated cells. Error bar = 100 μm. ****p < 0.0001 (right panel). **C** Migration and invasion assays of SW480-FAP1 and SW480-shFAP2 cells transfected with RELA mutation plasmid. Error bar = 100 μm. ****p < 0.0001. siNC: negative control siRNA; sip65: p65 knockdown siRNA; vector: empty vector; RELA mutation: RELA(S536D) mutation vector.

It has been reported that NF-κB pathway play important roles in carcinogenesis^22^. We speculated the FAP exerted pro-metastatic function dependent on NF-κB pathway. When siRNA was used to knock down the p65 transcript, the migration and invasion activity, which was stimulated by FAP overexpression treatment, was partially reversed (Fig. 5A, B). A transcriptional inhibitor of p65, JSH-23, decreased the number of migrating or invading HCT116-FAP and HCT8-FAP cells (Supplemental Fig. 5A, B). A mutated p65 (S536D), imitating the phosphorylation of p65, was synthetized. Activation of p65 increased the number of migrating and invading SW480-shFAP and DLD1-shFAP cells (Fig. 5C and Supplemental Fig. 5C). All the above results showed that FAP promoted CRC metastasis dependent on NF-κB signaling pathway.

### The soluble FAP promoted CRC cell metastasis

**Fig. 6:**
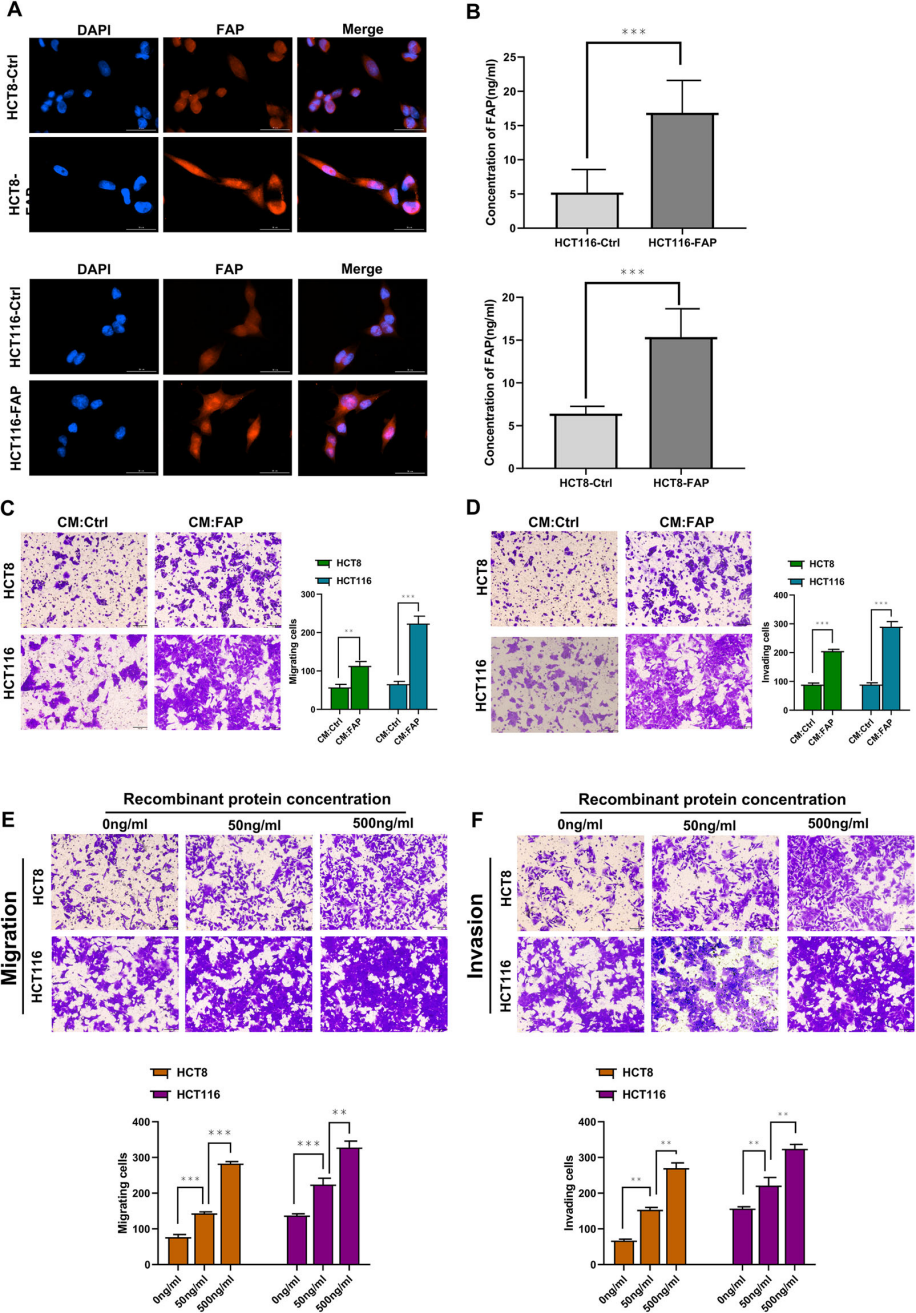
The soluble FAP promoted CRC cell metastasis. **A** Immunofluorescence analysis of FAP location in HCT116-FAP and HCT8-FAP cells. Error bar = 30 μm. **B** ELISA assay detects the concentration of FAP in media of HCT116-FAP, HCT8-FAP, HCT116-Ctrl, and HCT8-Ctrl cells. Ctrl: control; FAP: FAP overexpression. **C** Migration assays of HCT116 and HCT8 cells treated with control medium and FAP-overexpressed medium. Error bar = 100 μm. *p < 0.05, ***p < 0.001. The CM was added into the upper chamber. CM: conditioned medium. **D** Invasion assays of HCT116 and HCT8 cells treated with control medium and FAP-overexpressed medium. Error bar = 100 μm. *p < 0.05, ***p < 0.001. The CM was added into the upper chamber. **E** Migration assays of HCT116 and HCT8 cells treated with different concentration of recombinant FAP. Error bar = 100 μm. **p < 0.01, ***p < 0.001. **F** Invasion assays of HCT116 and HCT8 cells treated with different concentration of recombinant FAP. Error bar = 100 μm. **p < 0.01, ***p < 0.001.

Previous works have shown that FAP could be secreted into circulation^23^. Immunofluorescence showed the majority of FAP was dispersely distributed in CRC cytoplasm (Fig. 6A). ELISA assay showed higher concentration of FAP in the medium of HCT116-FAP and HCT8-FAP cells compared to control cells (Fig. 6B). These data indicated that FAP could be secreted by cancer cells. To investigate whether soluble FAP promotes metastasis, we collected CM from control cells and FAP-overexpressed cells. CM from FAP-overexpressed cells promoted the migration and invasion of HCT116 and HCT8 cells (Fig. 6C, D). We speculated that soluble FAP could promote metastasis. The recombinant FAP promoted migration and invasion of HCT116 and HCT8 cells in a dose-dependent manner (Fig. 6E, F). The recombinant FAP activated the NF-κB signaling pathway (Supplemental Fig. 6A, B). These results showed that soluble FAP promoted CRC cell metastasis.

### FAP binds ENO1 to promote metastasis and activated NF-κB signaling pathway

**Fig. 7:**
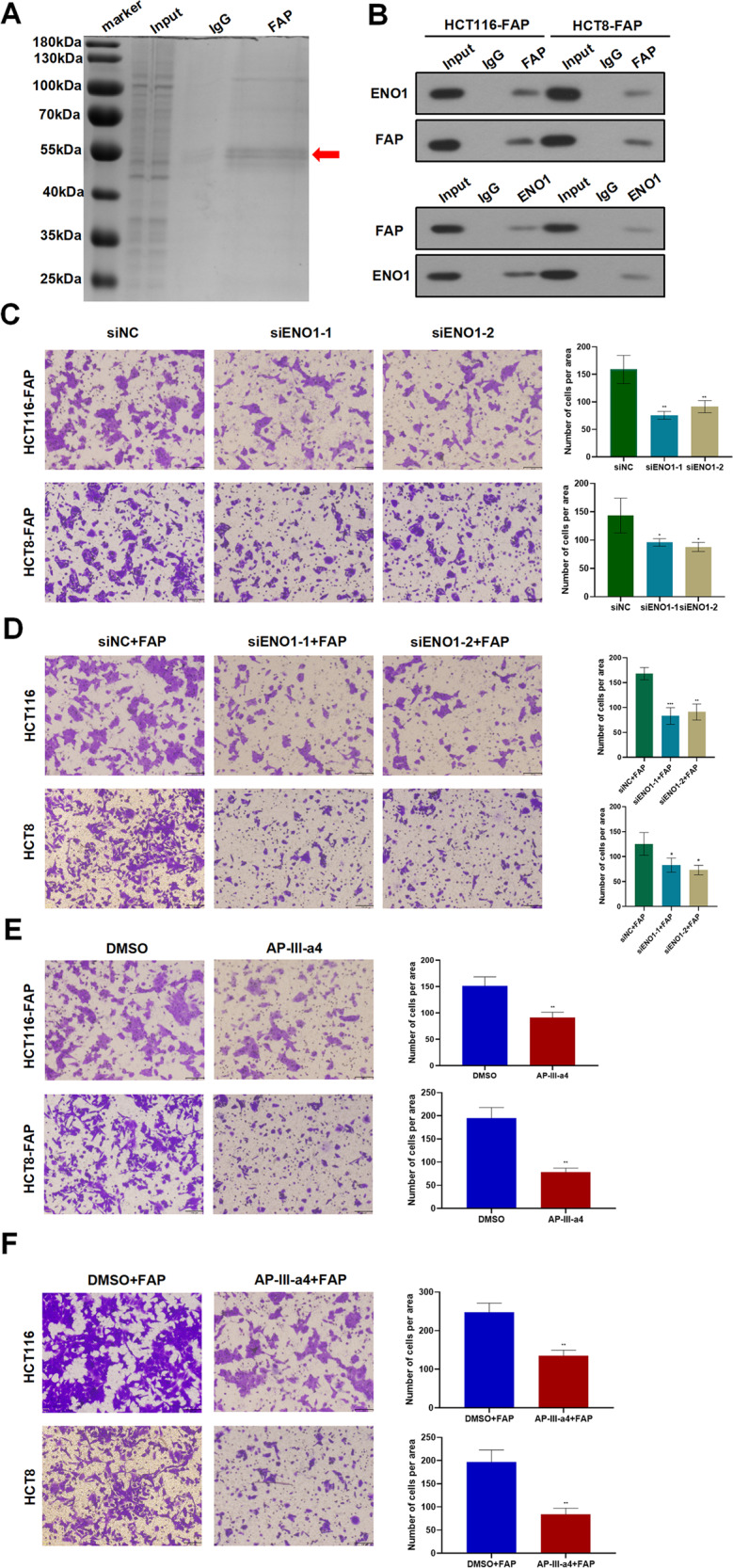
FAP binds ENO1 to promote metastasis and activate the NF-κB signaling pathway. **A** Silver staining of the immunoprecipitates from HCT116 cell lysates in IgG and anti-FAP group. Input: total cell lysates; IgG: cell lysates immunoprecipitated by negative control IgG; FAP: cell lysates immunoprecipitated by anti-FAP antibody. **B** Western blot analysis of indicated proteins in immunoprecipitates of HCT116-FAP and HCT8-FAP cells. Input: total cell lysates; IgG: cell lysates immunoprecipitated by negative control IgG; FAP: cell lysates immunoprecipitated by anti-FAP antibody. ENO1: cell lysates immunoprecipitated by anti-ENO1 antibody. **C** Migration assays of HCT116-FAP and HCT8-FAP cells after ENO1 is knocked down. siNC: negative control siRNA; siENO1: ENO1 siRNA. Error bar = 100 μm. *p < 0.05, **p < 0.01. **D** Migration assays of indicated cells treated with 50 ng/ml recombinant FAP. siNC: negative control siRNA; siENO1: ENO1 siRNA. Error bar = 100 μm. *p < 0.05. **E** Migration assays of indicated cells treated with AP-III-a4. Error bar = 100 μm. **p < 0.01. **F** Migration assays of indicated cells treated with AP-III-a4 and 50 ng/ml recombinant FAP. Error bar = 100 μm. **p < 0.01.

Secreted proteins often interact with the membrane receptor to assert its function. However, the proteins interacted with FAP are poorly understood. Immunoprecipitation assays were performed to explore the FAP-interacted proteins. Silver staining assay showed a band located at 55 kd (Fig. 7A). Then, we performed LC/MS assay to identify the proteins interacted with FAP (Supplemental Table 3). Among these proteins, ENO1 is a membrane protein and has been reported to have a close relationship with NF-κB signaling pathway^24,25^. Co-immunoprecipitation assay confirmed FAP bound to ENO1 in HCT116 and HCT8 cells (Fig. 7B), whereas levels of FAP had no effect on mRNA and protein levels of ENO1 (Supplemental Fig. 7A, B). We speculated that FAP bound to ENO1, to activate the NF-κB signaling pathway and promote metastasis. Knockdown of ENO1 attenuated migration and invasion of HCT116-FAP and HCT8-FAP cells (Fig. 7C and Supplemental Fig. 7D), and partially inactivated NF-κB signaling pathway (Supplemental Fig. 7H). Recombinant FAP failed to promote migration and invasion of HCT116 and HCT8 cells, and to activate NF-κB signaling pathway when ENO1 was knocked down (Fig. 7D and Supplemental Fig. 7E, I). AP-III-a4 is a non-substrate analog that directly binds to enolase and inhibits its function independent of enzymatic activity, which has been widely used in elucidating the role of ENO1 in many kinds of tumors^24,26,27^. AP-III-a4 decreased the number of HCT116-FAP and HCT8-FAP cells, and inactivated NF-κB signaling pathway (Fig. 7E and Supplemental Fig. 7F, J). Treated with AP-III-a4, recombinant FAP failed to promote migration and invasion, and to activate NF-κB signaling pathway (Fig. 7F and Supplemental Fig. 7G, K). These works showed that FAP binds to ENO1, to activate the NF-κB signaling pathway.

Next, we were intended to explore whether FAP had an effect on the expression of membrane ENO1. After isolating the membrane parts and cytoplasmic part of cells, neither cell surface ENO1 nor cytosolic ENO1 were not influenced by FAP knockdown (Supplemental Fig. 8A, B). Then, we speculated that soluble FAP is possible to bind to membrane ENO1. The cell lines were treated with 2 μg/ml recombinant FAP protein in the serum-free medium for 8 h. After membrane and cytoplasmic protein were isolated, immunoprecipitation assay showed that FAP bound to membrane ENO1 instead of cytoplasmic ENO1 (Supplemental Fig. 8C, D).

### ENO1 is upregulated and correlates with clinical outcomes in CRC patients

**Fig. 8:**
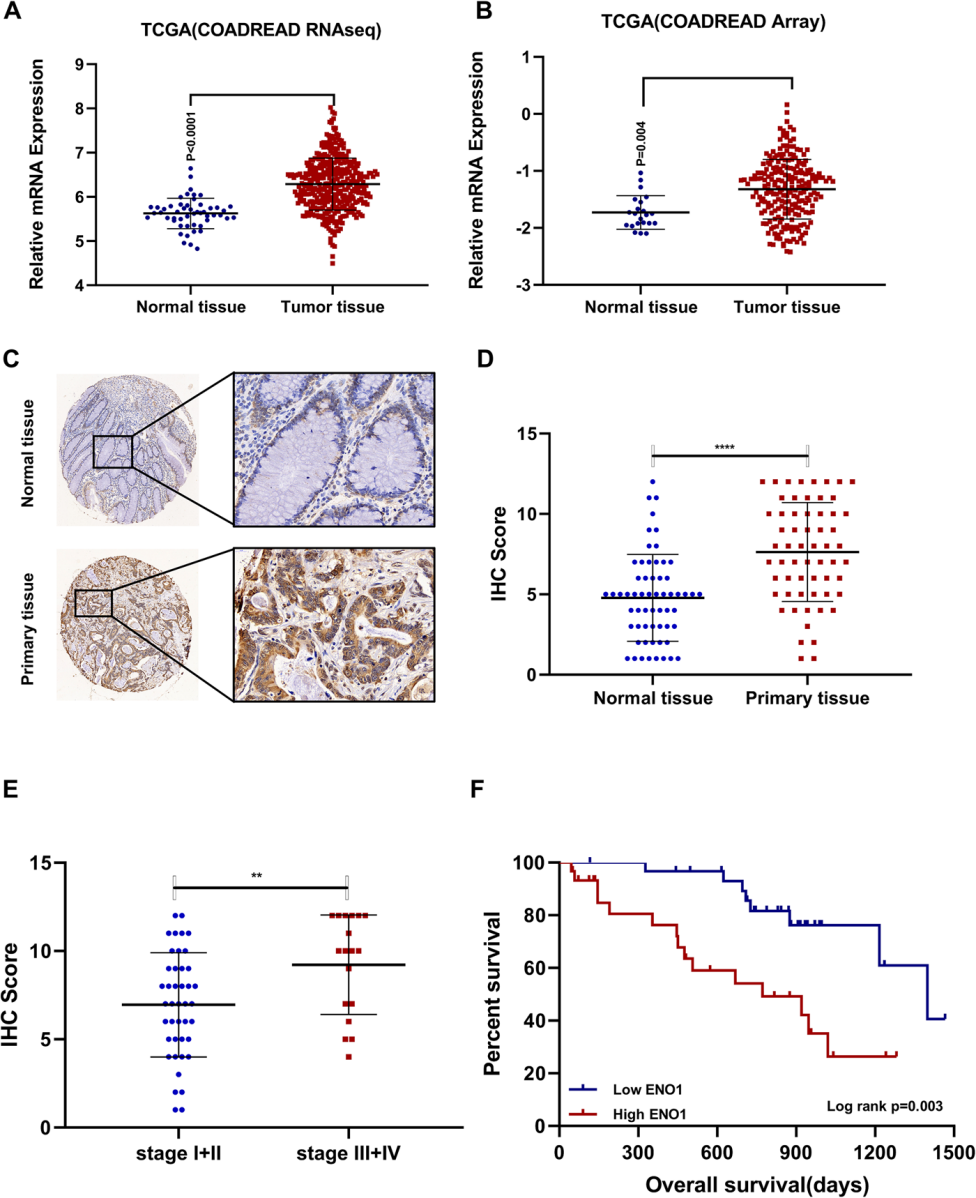
ENO1 is upregulated and correlates with clinical outcomes in CRC patients. **A** ENO1 mRNA expression pattern based on RNA-sequencing data in TCGA. **B** ENO1 mRNA expression pattern based on microarray data in TCGA. **C** Representative images of ENO1 staining in cancer tissue and normal tissue. **D** Comparision of ENO1 IHC score between tumor tissue and normal tissue. ****p < 0.001. **E** Comparision of ENO1 IHC score between stage I + II and stage III + IV patients. **p < 0.01. **F** Kaplan–Meier analysis of ENO1 high-expression group and ENO1-low-expression group. Median IHC score was used to stratify high- and low-expression group, to analyze OS and RFS.

To examine the expression pattern of ENO1, we analyzed the RNA sequencing and microarray data in TCGA. Compared to normal tissue, ENO1 was upregulated in tumor tissue (Fig. 8A, B). Results from immunohistochemistry showed that ENO1 protein was upregulated in tumor tissue (Fig. 8C) and levels of ENO1 was also upregulated in late-stage CRC (Fig. 8D). Kaplan–Meier analysis showed that high ENO1 levels were associated with shorter overall survival (Fig. 8E). These data indicated that ENO1 level could be a potential biomarker in CRC patients.

## Discussion

Cancer is a disease involving complicated reciprocal networks between cancer cells and stromal cells^28^. FAP is a marker of CAFs, which are proved to be an important contributor to carcinogenesis. Although current researches have shown that FAP is not restricted to stromal cells, FAP has been detected in mature adipocytes^7^, glucagon-positive α-cells in the pancreas^8^, astrocytes^9^, and M2 macrophages^29^. In addition, FAP has been found in cancer cells, such as in lung cancer cell cytoplasma, by immunohistochemistry^30^. Using immunohistochemistry, FAP was also found to be expressed in breast cancer cells^31–33^, in lung cancer cells^34^, and in pancreatic cancer cells^35,36^. Using flow cytometry, FAP has been found in a subset of pancreatic cancer cell^37^. Kahounová et al.^38^ and Wolczyk et al.^39^ have reported that FAP was expressed in breast cancer cell lines and in prostate cancer cell lines. The expression pattern in cancer cells has been confirmed in Human Protein Atlas Database with regard to CRC cells (https://www.proteinatlas.org/ENSG00000078098-FAP/pathology/colorectal+cancer), breast cancer cells (https://www.proteinatlas.org/ENSG00000078098-FAP/pathology/breast+cancer), and pancreatic cancer cells (https://www.proteinatlas.org/ENSG00000078098-FAP/pathology/pancreatic+cancer). Besides, FAP expression is higher compared to normal colon epithelial cells, but in parallel with the expression in CAFs. FAP acts as an oncogene in multiple cancer cells. Knockdown of FAP in oral squamous cell carcinoma cells reduce the migration and invasion through Matrigel^40^. Consistent with our results, we found FAP promoted CRC cell migration and invasion. Previous researches have shown FAP has an indirect effect on tumor cells. Recombinant FAP added to the culture medium promotes the resistance of ovarian cancer cell to cisplatin^41^. Co-culture of cancer cells with FAP^+^ CAFs promote migration and invasion, which could be reduced by anti-FAP antibody in lung cancer cells^42^. Consistent with previous works, we found FAP could be secreted by tumor cells and promote the cancer cell migration and invasion.

NF-κB signaling pathway plays an important role in metastasis and is activated in various cancers including CRC^22^. Our results showed that NF-κB signaling pathway was activated and was essential for FAP-mediated metastasis. A positive correlation between FAP and NF-κB signaling pathway was observed in clinical samples. Recovery assay confirmed FAP promoted metastasis dependent on NF-κB signaling pathway. Overexpression of ENO1 could activate NF-κB signaling pathway^43–45^. ENO1 promotes the cell growth and migration through NF-κB signaling pathway in the glioma^25^. Ligand interaction with ENO1 could also activate the NF-κB signaling pathway. Lee et al.^24^ has reported that ApoB binds to ENO1 inducing a proinflammatory response in rheumatoid arthritis. Similarly, we are the first to identify that FAP binds to ENO1, to activate NF-κB signaling pathway and induce inflammatory gene expression in CRC without an effect on expression of ENO1 mRNA or protein.

FAP and ENO1 are potential biomarkers for CRC patients. The levels of FAP and ENO1 in cancer tissues could predict the overall survival and recurrence-free survival in patients. Besides, the levels of FAP mRNA are increasing with the development of cancer progression. Larrinaga and colleagues^46^ have reported that FAP expression in cancer tissues is associated with CRC lymph node metastasis and liver metastasis. In other types of tumors, high levels of FAP in tumors are associated with poor survival^47–49^, and in lung cancer, ENO1 is a biomarker associated with patient survival^50^. Our data show FAP and ENO1 could be a potential biomarker of CRC patients.

In summary, this study is first to identify that FAP binds to ENO1, to exert a pro-metastatic effect in CRC. FAP dependent on ENO1 activates intercellular NF-κB signaling pathway to induce a proinflammatory response in CRC cells. High level of FAP and ENO1 expression predicted a worse clinical outcome. Taken together, our results comprehensively illustrate function and mechanism of FAP and may provide a novel strategy for the treatment of CRC patients.

## Supplementary information

Supplementary Figure Legend

supplemental figure 1

supplemental figure 2

supplemental figure 3

supplemental figure 4

supplemental figure 5

supplemental figure 6

supplemental figure 7

supplemental figure 8

supplemental file 1

supplemental table 1

supplemental table 2

supplemental table 3

## References

[1] Siegel RL (2017). Colorectal cancer statistics, 2017. CA Cancer J. Clin..

[2] Zhan HX (2017). Crosstalk between stromal cells and cancer cells in pancreatic cancer: New insights into stromal biology. Cancer Lett..

[3] Hanahan D, Weinberg RA (2011). Hallmarks of cancer: the next generation. Cell.

[4] Unterleuthner, D. et al. Cancer-associated fibroblast-derived WNT2 increases tumor angiogenesis in colon cancer. *Angiogenesis* **23**, 159–177 (2019). 10.1007/s10456-019-09688-8 PMC7160098 31667643

[5] Pelon F (2020). Cancer-associated fibroblast heterogeneity in axillary lymph nodes drives metastases in breast cancer through complementary mechanisms. Nat. Commun..

[6] Yang X (2016). FAP promotes immunosuppression by cancer-associated fibroblasts in the tumor microenvironment via STAT3-CCL2 signaling. Cancer Res..

[7] Lessard J (2015). Characterization of dedifferentiating human mature adipocytes from the visceral and subcutaneous fat compartments: fibroblast-activation protein alpha and dipeptidyl peptidase 4 as major components of matrix remodeling. PLoS ONE.

[8] Busek P, Hrabal P, Fric P, Sedo A (2015). Co-expression of the homologous proteases fibroblast activation protein and dipeptidyl peptidase-IV in the adult human Langerhans islets. Histochem. Cell Biol..

[9] Busek P (2016). Fibroblast activation protein alpha is expressed by transformed and stromal cells and is associated with mesenchymal features in glioblastoma. Tumour Biol..

[10] Liu J (2018). Stromal fibroblast activation protein alpha promotes gastric cancer progression via epithelial-mesenchymal transition through Wnt/ beta-catenin pathway. BMC Cancer.

[11] da Silva AC, Jammal MP, Etchebehere RM, Murta EFC, Nomelini RS (2018). Role of alpha-smooth muscle actin and fibroblast activation protein alpha in ovarian neoplasms. Gynecol. Obstet. Invest.

[12] Wang M (2018). Long non-coding RNA H19 confers 5-Fu resistance in colorectal cancer by promoting SIRT1-mediated autophagy. Cell Death Dis..

[13] Wan L (2019). SRSF6-regulated alternative splicing that promotes tumour progression offers a therapy target for colorectal cancer. Gut.

[14] Zhuo W (2019). Long noncoding RNA GMAN, upregulated in gastric cancer tissues, is associated with metastasis in patients and promotes translation of Ephrin A1 by competitively binding GMAN-AS. Gastroenterology.

[15] Shen SM (2019). PTENalpha and PTENbeta promote carcinogenesis through WDR5 and H3K4 trimethylation. Nat. Cell Biol..

[16] Yang G (2016). FCN2 inhibits epithelial-mesenchymal transition-induced metastasis of hepatocellular carcinoma via TGF-beta/Smad signaling. Cancer Lett..

[17] Krepela E, Busek P, Hilser M, Vanickova Z, Sedo A (2017). Species-specific real-time RT-PCR analysis of expression of stromal cell genes in a tumor xenotransplantation model in mice. Biochem. Biophys. Res. Commun..

[18] Tyulkina DV, Pleshkan VV, Alekseenko IV, Kopantseva MR, Sverdlov ED (2016). Expression of the FAP gene in non-fibroblast human cell lines. Development of cancer-associated fibroblast models. Dokl. Biochem. Biophys..

[19] Augoff K (2014). Upregulated expression and activation of membraneassociated proteases in esophageal squamous cell carcinoma. Oncol. Rep..

[20] Tojima Y (2000). NAK is an IkappaB kinase-activating kinase. Nature.

[21] Darnell JE (1997). STATs and gene regulation. Science.

[22] Baud V, Karin M (2009). Is NF-kappaB a good target for cancer therapy? Hopes and pitfalls. Nat. Rev. Drug Discov..

[23] Pure E, Blomberg R (2018). Pro-tumorigenic roles of fibroblast activation protein in cancer: back to the basics. Oncogene.

[24] Lee JY (2018). Apolipoprotein B binds to enolase-1 and aggravates inflammation in rheumatoid arthritis. Ann. Rheum. Dis..

[25] Song Y (2014). Alpha-enolase as a potential cancer prognostic marker promotes cell growth, migration, and invasion in glioma. Mol. Cancer.

[26] Chen W (2020). A bioenergetic shift is required for spermatogonial differentiation. Cell Discov..

[27] Zheng ZG (2019). Inhibition of HSP90beta improves lipid disorders by promoting mature SREBPs degradation via the ubiquitin-proteasome system. Theranostics.

[28] Ishii G, Ochiai A, Neri S (2016). Phenotypic and functional heterogeneity of cancer-associated fibroblast within the tumor microenvironment. Adv. Drug Deliv. Rev..

[29] Arnold JN, Magiera L, Kraman M, Fearon DT (2014). Tumoral immune suppression by macrophages expressing fibroblast activation protein-alpha and heme oxygenase-1. Cancer Immunol. Res..

[30] Du H, Chen D, Zhou Y, Han Z, Che G (2014). Fibroblast phenotypes in different lung diseases. J. Cardiothorac. Surg..

[31] Park SY, Kim HM, Koo JS (2015). Differential expression of cancer-associated fibroblast-related proteins according to molecular subtype and stromal histology in breast cancer. Breast Cancer Res. Treat..

[32] Park CK, Jung WH, Koo JS (2016). Expression of cancer-associated fibroblast-related proteins differs between invasive lobular carcinoma and invasive ductal carcinoma. Breast Cancer Res. Treat..

[33] Jung YY, Lee YK, Koo JS (2015). Expression of cancer-associated fibroblast-related proteins in adipose stroma of breast cancer. Tumour Biol..

[34] Kraman M (2010). Suppression of antitumor immunity by stromal cells expressing fibroblast activation protein-alpha. Science.

[35] Kawase T (2015). Fibroblast activation protein-alpha-expressing fibroblasts promote the progression of pancreatic ductal adenocarcinoma. BMC Gastroenterol..

[36] Busek P (2016). Increased tissue and circulating levels of dipeptidyl peptidase-IV enzymatic activity in patients with pancreatic ductal adenocarcinoma. Pancreatology.

[37] Lo A., et al. Fibroblast activation protein augments progression and metastasis of pancreatic ductal adenocarcinoma. *JCI Insight* **2**, e92232 (2017). 10.1172/jci.insight.92232 PMC5841864 28978805

[38] Kahounova Z (2018). The fibroblast surface markers FAP, anti-fibroblast, and FSP are expressed by cells of epithelial origin and may be altered during epithelial-to-mesenchymal transition. Cytom. A.

[39] Wolczyk D (2016). TNF-alpha promotes breast cancer cell migration and enhances the concentration of membrane-associated proteases in lipid rafts. Cell Oncol..

[40] Wang H (2014). Downregulation of FAP suppresses cell proliferation and metastasis through PTEN/PI3K/AKT and Ras-ERK signaling in oral squamous cell carcinoma. Cell Death Dis..

[41] Mhawech-Fauceglia P (2015). Stromal expression of fibroblast activation protein alpha (FAP) predicts platinum resistance and shorter recurrence in patients with epithelial ovarian cancer. Cancer Microenviron..

[42] Teichgraber V (2015). Specific inhibition of fibroblast activation protein (FAP)-alpha prevents tumor progression in vitro. Adv. Med. Sci..

[43] Bae S (2012). alpha-Enolase expressed on the surfaces of monocytes and macrophages induces robust synovial inflammation in rheumatoid arthritis. J. Immunol..

[44] Pastor MD (2013). Identification of proteomic signatures associated with lung cancer and COPD. J. Proteom..

[45] Choi J (2015). The anti-inflammatory effect of GV1001 mediated by the downregulation of ENO1-induced pro-inflammatory cytokine production. Immune Netw..

[46] Solano-Iturri JD (2020). Altered expression of fibroblast activation protein-alpha (FAP) in colorectal adenoma-carcinoma sequence and in lymph node and liver metastases. Aging.

[47] Hu M, Qian C, Hu Z, Fei B, Zhou H (2017). Biomarkers in tumor microenvironment? Upregulation of fibroblast activation protein-alpha correlates with gastric cancer progression and poor prognosis. OMICS.

[48] Yuan D (2013). Overexpression of fibroblast activation protein and its clinical implications in patients with osteosarcoma. J. Surg. Oncol..

[49] Shi J (2020). The prognostic significance of fibroblast activation protein-alpha in human lung adenocarcinoma. Ann. Transl. Med..

[50] Khanmohammadi A (2020). Electrochemical biosensors for the detection of lung cancer biomarkers: a review. Talanta.

