# Supplementary material for: Colorectal cancer cell intrinsic fibroblast activation protein alpha binds to Enolase1 and activates NF-κB pathway to promote metastasis
Source: Cell Death Dis. 2021 May 25;12(6):543. doi: 10.1038/s41419-021-03823-4 (PMC8149633; doi:10.1038/s41419-021-03823-4)
Supplement: Supplementary file 1 — Supplementary Figure Legend [file 41419_2021_3823_MOESM1_ESM.docx]

**Supplemental Figure Legend 1. FAP is upregulated in CRC cells and associated with poor clinical outcomes.**

**A.** FAP mRNA expression pattern based on RNA sequencing data in TCGA. **B.** FAP mRNA expression pattern based on microarray data in TCGA. **C.** The mRNA Expression of FAP in ovarian cancer cells and normal epithelia. The mRNA expression was acquired from dataset (GSE14407) in Gene Expression Omnibus. The cancer epithelia and normal epithelia were isolated by LCM method. **D.** Western blot analysis of FAP in cancer cells and cancer-associated fibroblasts isolated by MACS.CC: cancer cell; CAF: cancer-associated fibroblast.

**Supplemental Figure Legend 2. FAP promotes CRC cell migration and invasion *in vitro*.**

**A.** qPCR analysis of FAP level in various CRC cell lines. **B.** qPCR analysis of FAP-mRNA level in FAP over-expressed and FAP knock-down cells. **C.** CCK-8 assay of indicated cell lines with the over-expression of FAP and knock-down of FAP. **D.** Colony formation assay of indicated cell lines with the over-expression of FAP and knock-down of FAP. shNC: negative control shRNA; shFAP: FAP knock-down shRNA.

**Supplemental Figure Legend 3. FAP promotes CRC cell migration and invasion *in vivo.***

FAP mRNA expression levels in GSE161097 and GSE51244 datasets from Gene Expression Omnibus. Left panel: Primary tumor and peritoneal tumor were the primary lesion and peritoneal metastatic acquired from the same patients; right panel: primary tumor and lung metastatic lesion were acquired from different patients. **p*<0.05, ***p*<0.01.

**Supplemental Figure Legend 4. FAP exerted its function dependent on NF-κB pathway.**

**A.** Volcano plot showed different expressed genes in SW480-shFAP1 and SW480-shFAP2 cells compared to SW480-shNC. **B.** GO analysis of different expressed genes. The arrow pointed to the GO function evolved in further investigation. **C.** Western blot analysis of the proteins in NF-κB and STAT3 pathway in indicated groups. Ctrl: negative control; FAP: FAP over-expression; shNC: negative control shRNA; shFAP: FAP shRNA. **D.** qPCR assay detected mRNA of NF-κB and STAT3 pathway related genes in DLD1 FAP knock down and HCT8 FAP over-expressed cells. **p*<0.05, ***p*<0.01, *** *p*<0.001.

**Supplemental Figure Legend 5. FAP exerted its function dependent on NF-κB pathway.**

**A.** Migration and invasion assays of HCT116-FAP cells treated with JSH-23. Error bar=100μm. **p*<0.05, ***p*<0.01. **B.** Migration and invasion assays of HCT8-FAP cells treated with JSH-23. Error bar=100μm. ****p*<0.001.**C.** Migration and invasion assays of DLD1-shFAP1 and DLD1-shFAP2 cells transfected with RELA mutation plasmid. Error bar=100μm. ****p*<0.001.

**Supplemental Figure Legend 6. The soluble FAP promoted CRC cell metastasis.**

**A.** Western blot analysis of the proteins in NF-κB and STAT3 pathway in indicated groups after FAP 50ng/ml recombinant FAP treatment. **B.** qPCR assay detected mRNA of NF-κB and STAT3 pathway related genes in HCT116 and HCT8 cells after 50ng/ml recombinant FAP treatment.

**Supplemental Figure Legend 7. FAP binds to ENO1 to promote metastasis and activated NF-κB signaling pathway.**

**A.** qPCR assay detected ENO1 mRNA levels in indicated groups. Ctrl: negative control; FAP: FAP over-expression; shNC: negative control shRNA; shFAP: FAP knock down shRNA. **B.** Western blot analysis of ENO1 protein levels in indicated groups. Ctrl: negative control; FAP: FAP over-expression; shNC: negative control shRNA; shFAP: FAP knock down shRNA. **C.** Western blot analysis of the efficiency in indicated groups after ENO1 knock-down. siNC: negative control siRNA; siENO1: ENO1 knock down siRNA. **D.** Invasion assays of HCT116-FAP and HCT8-FAP cells after ENO1 is knockdown. Error bar=100μm. ****p*<0.001. siNC: negative control siRNA; siENO1: ENO1 knock down siRNA. **E.** Invasion assays of indicated cells treated with 50ng/ml recombinant FAP. Error bar=100μm. ****p*<0.001. siNC: negative control siRNA; siENO1: ENO1 knock down siRNA. **F.** Invasion assays of indicated cells treated with AP-III-a4. Error bar=100μm. ****p*<0.01, **p*<0.05. **G.** Invasion assays of indicated cells treated with AP-III-a4 and 50ng/ml recombinant FAP. Error bar=100μm. ****p*<0.01, **p*<0.05. **H.** Western blot analysis of the proteins in NF-κB and STAT3 pathway in indicated groups after ENO1 knock-down in HCT116-FAP and HC8-FAP cells. siNC: negative control siRNA; siENO1: ENO1 knock down siRNA. **I.** Western blot analysis of the proteins in NF-κB and STAT3 pathway in indicated groups after treatment of ENO1 knock-down and 50ng/ml recombinant FAP in HCT116and HCT8 cells. siNC: negative control siRNA; siENO1: ENO1 knock down siRNA. **J.** Western blot analysis of the proteins in NF-κB and STAT3 pathway in indicated groups after treatment of AP-III-a4 in HCT116-FAP and HC8-FAP cells. **K.** Western blot analysis of the proteins in NF-κB and STAT3 pathway in indicated groups after treatment of AP-III-a4 and 50ng/ml recombinant FAP and in HCT116 and HC8 cells.

**Supplemental Figure Legend 8. FAP binds to cell surface ENO1.**

**A & B**: western blot assay was applied to detected the ENO1 expression in membrane and cytoplasm in SW480 (A) and DLD1 (B) after FAP was knocked down. WCL: whole cell lysates. **C & D**: immunoprecipitation assay was applied to detected the interaction between ENO1 and FAP in membrane and cytoplasm of HCT116 (C) and HCT8 (D).
