# Supplementary figures and images for: Colorectal cancer cell intrinsic fibroblast activation protein alpha binds to Enolase1 and activates NF-κB pathway to promote metastasis
Source: Cell Death Dis. 2021 May 25;12(6):543. doi: 10.1038/s41419-021-03823-4 (PMC8149633; doi:10.1038/s41419-021-03823-4)

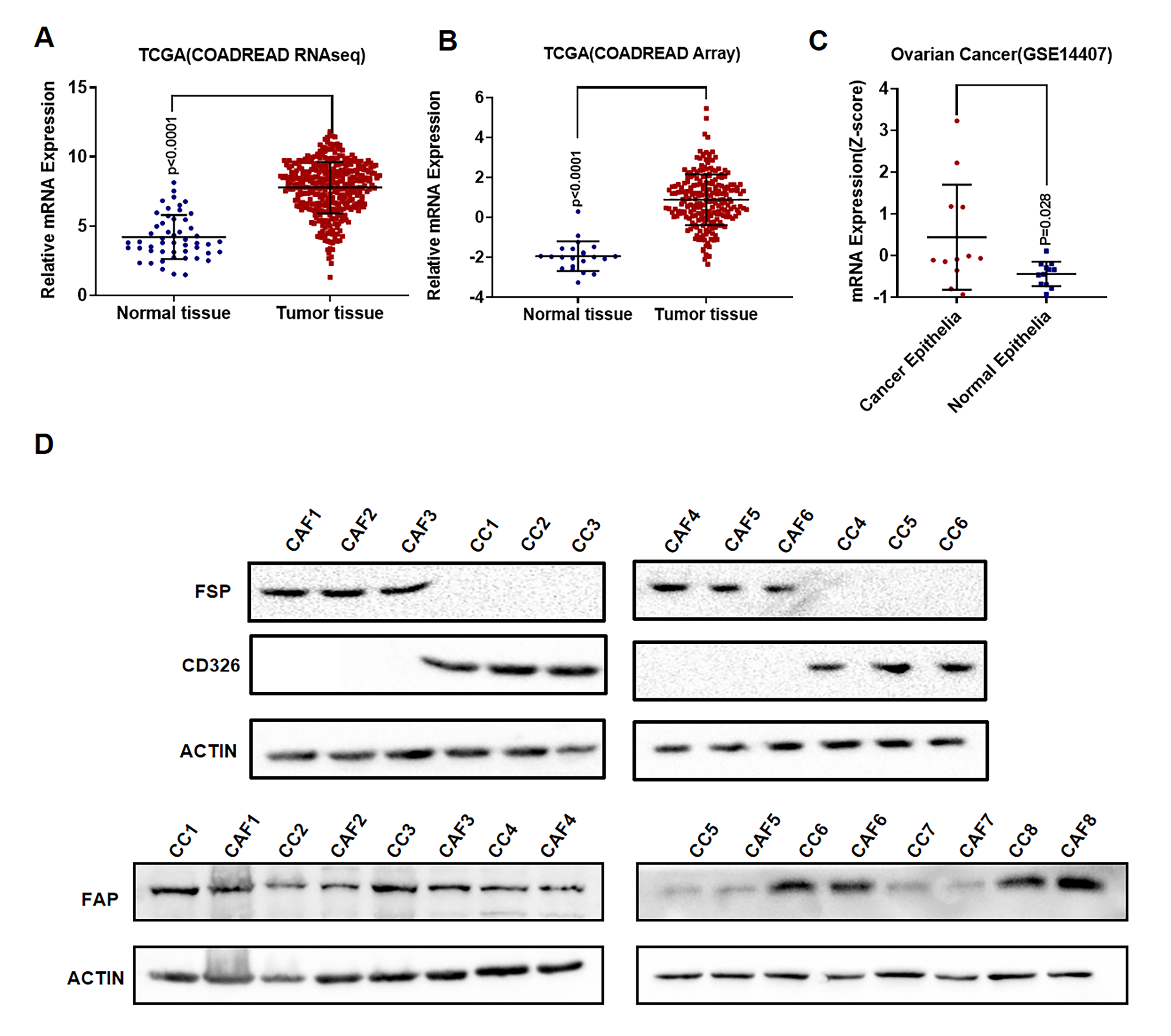

Supplement: Supplementary file 2 — supplemental figure 1 [file 41419_2021_3823_MOESM2_ESM.tif]

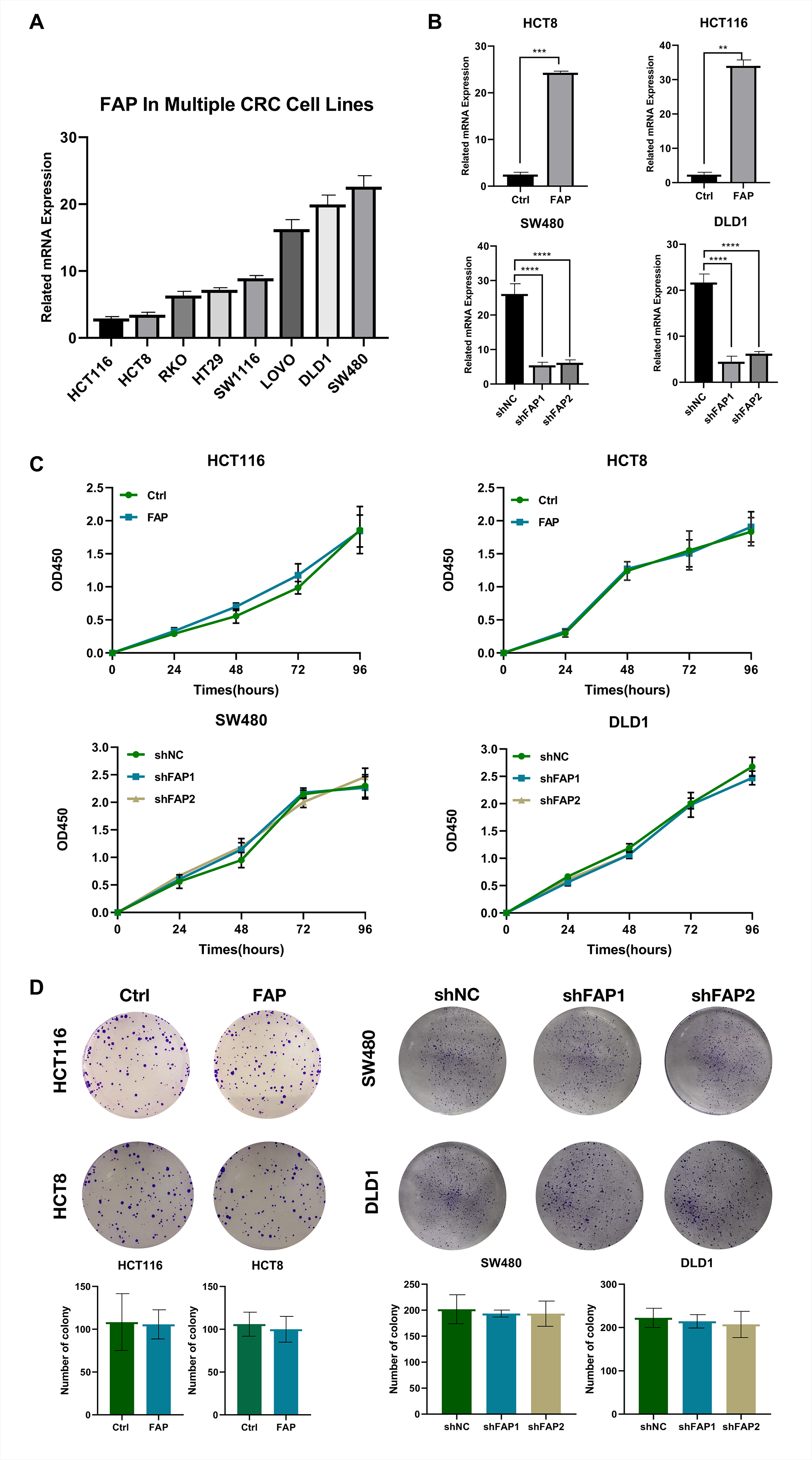

Supplement: Supplementary file 3 — supplemental figure 2 [file 41419_2021_3823_MOESM3_ESM.tif]

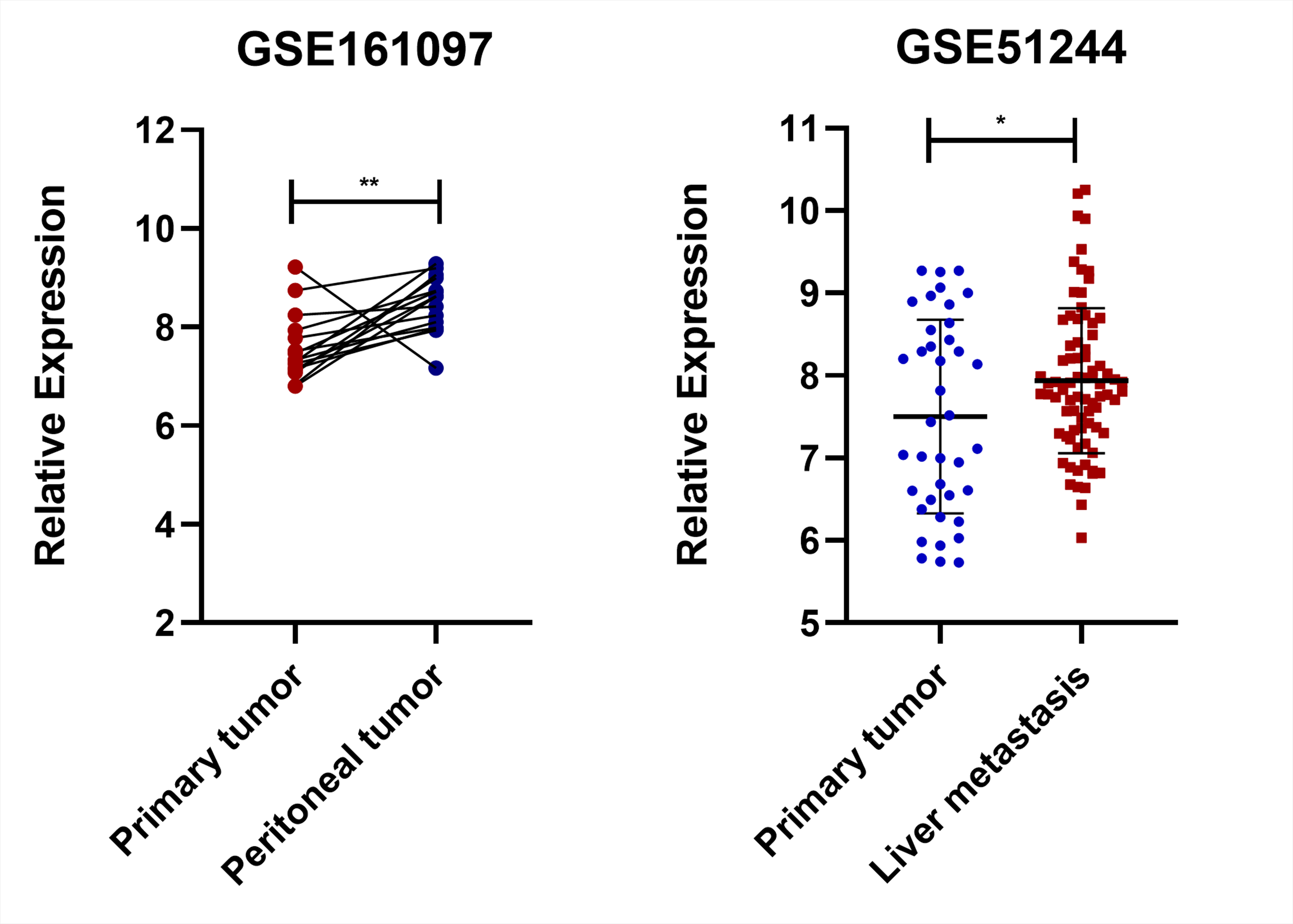

Supplement: Supplementary file 4 — supplemental figure 3 [file 41419_2021_3823_MOESM4_ESM.tif]

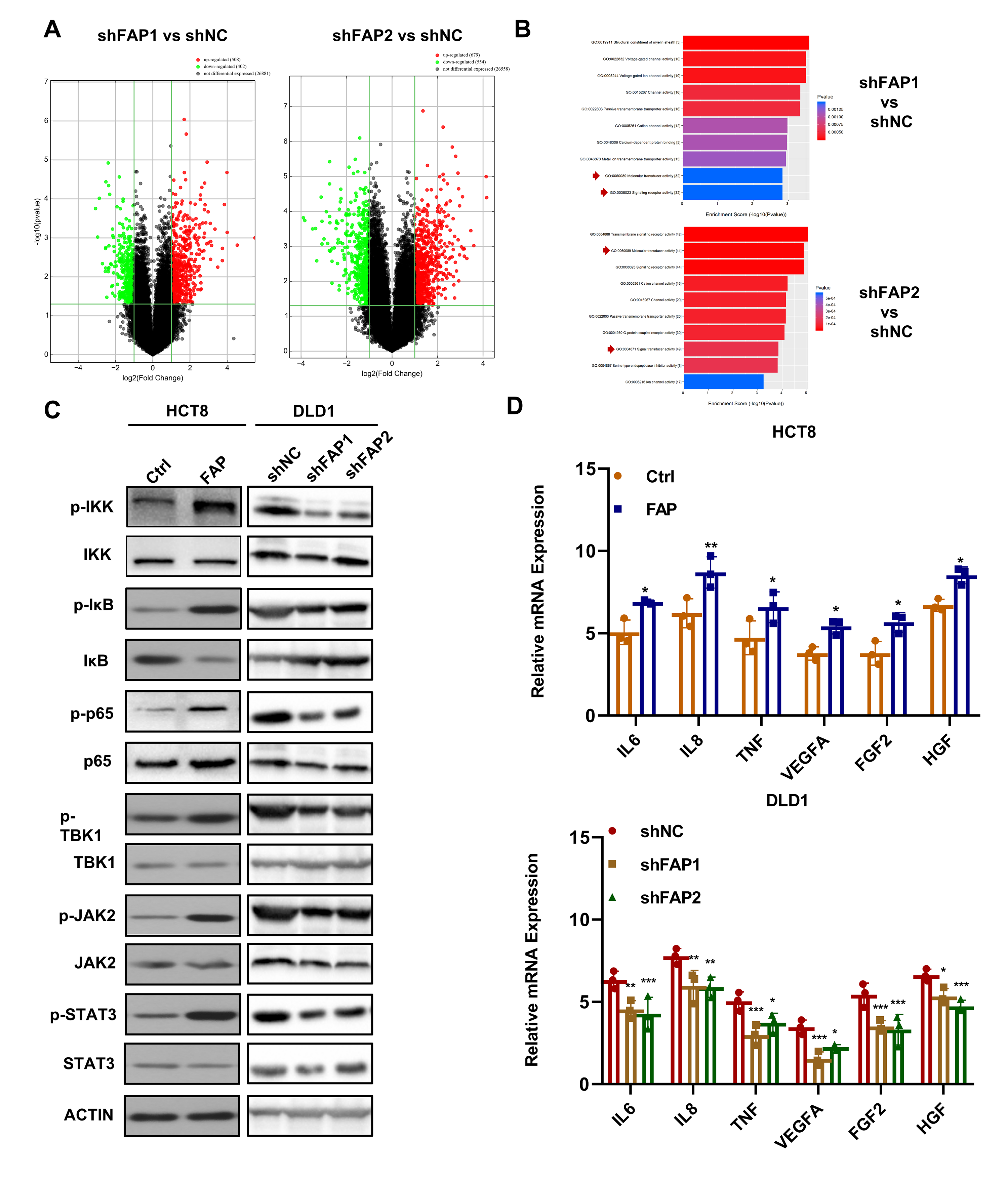

Supplement: Supplementary file 5 — supplemental figure 4 [file 41419_2021_3823_MOESM5_ESM.tif]

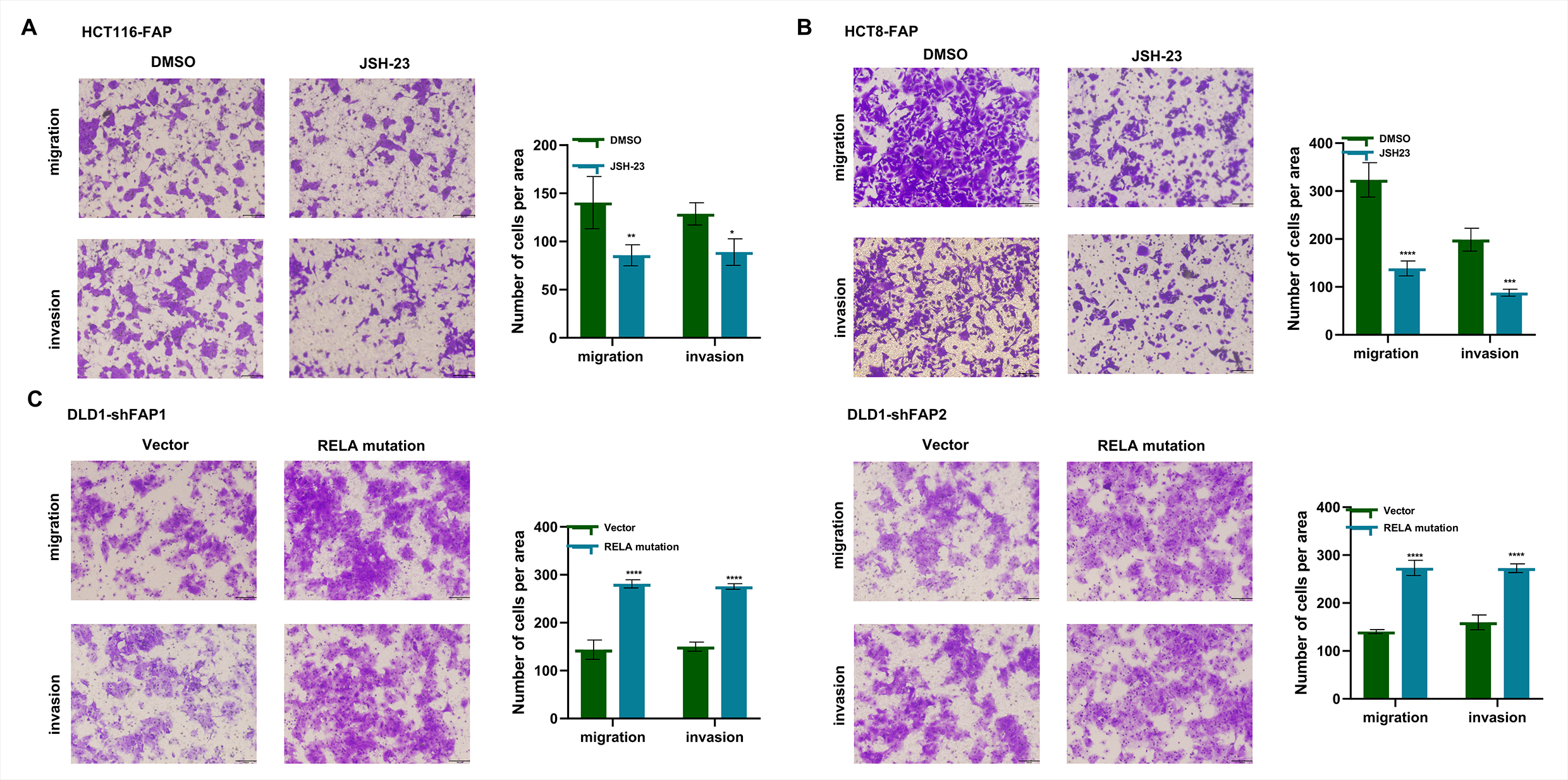

Supplement: Supplementary file 6 — supplemental figure 5 [file 41419_2021_3823_MOESM6_ESM.tif]

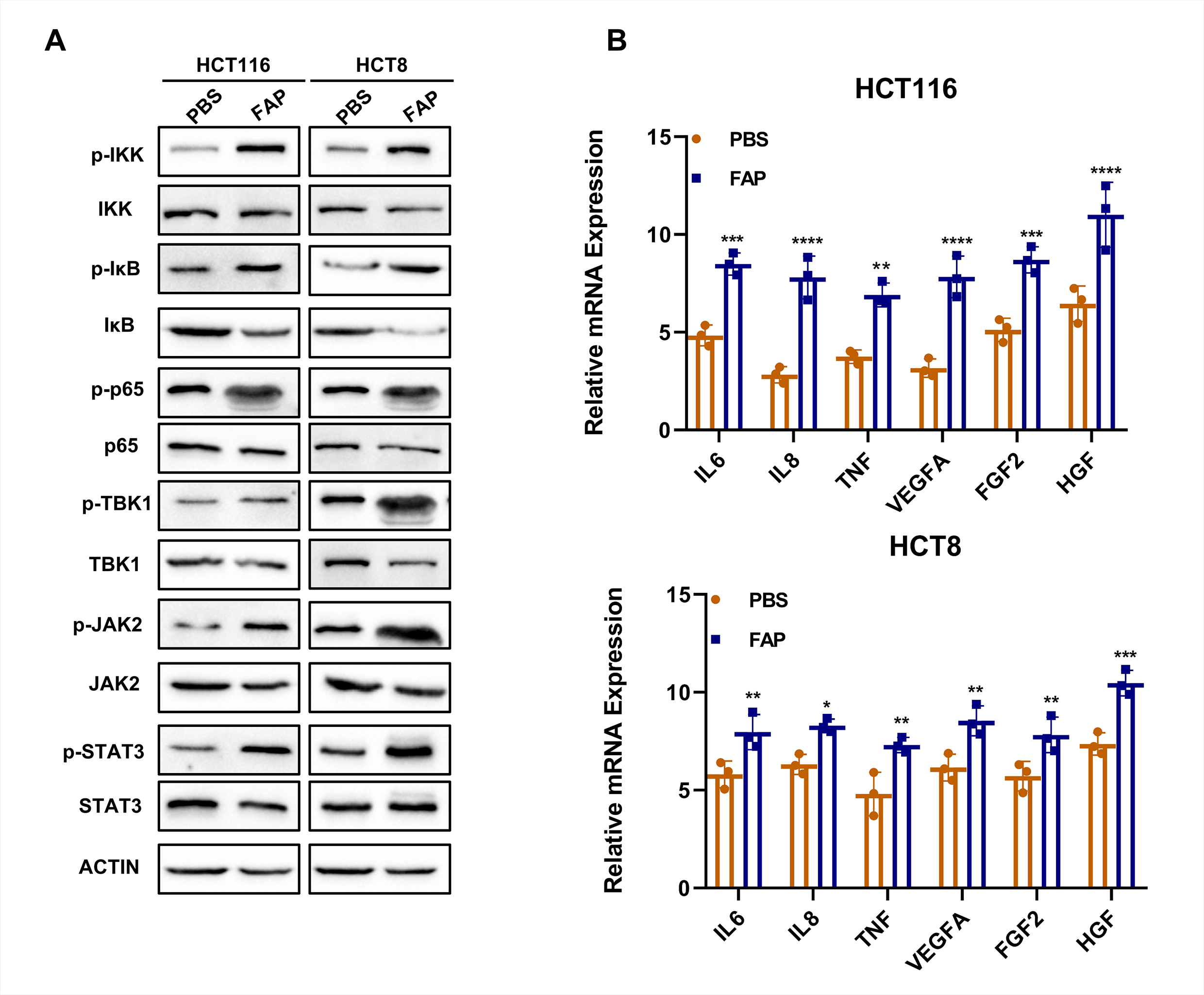

Supplement: Supplementary file 7 — supplemental figure 6 [file 41419_2021_3823_MOESM7_ESM.tif]

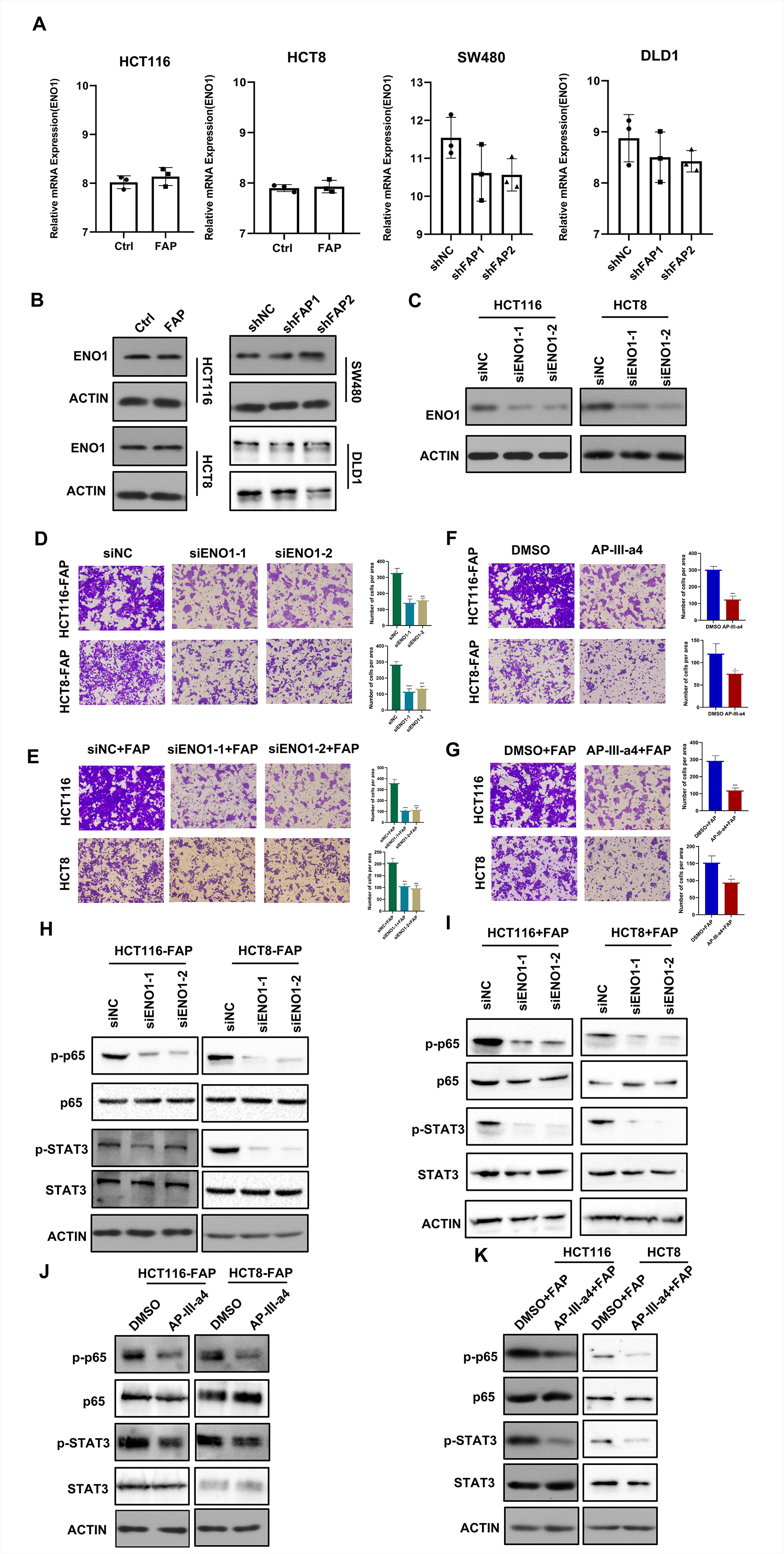

Supplement: Supplementary file 8 — supplemental figure 7 [file 41419_2021_3823_MOESM8_ESM.tif]

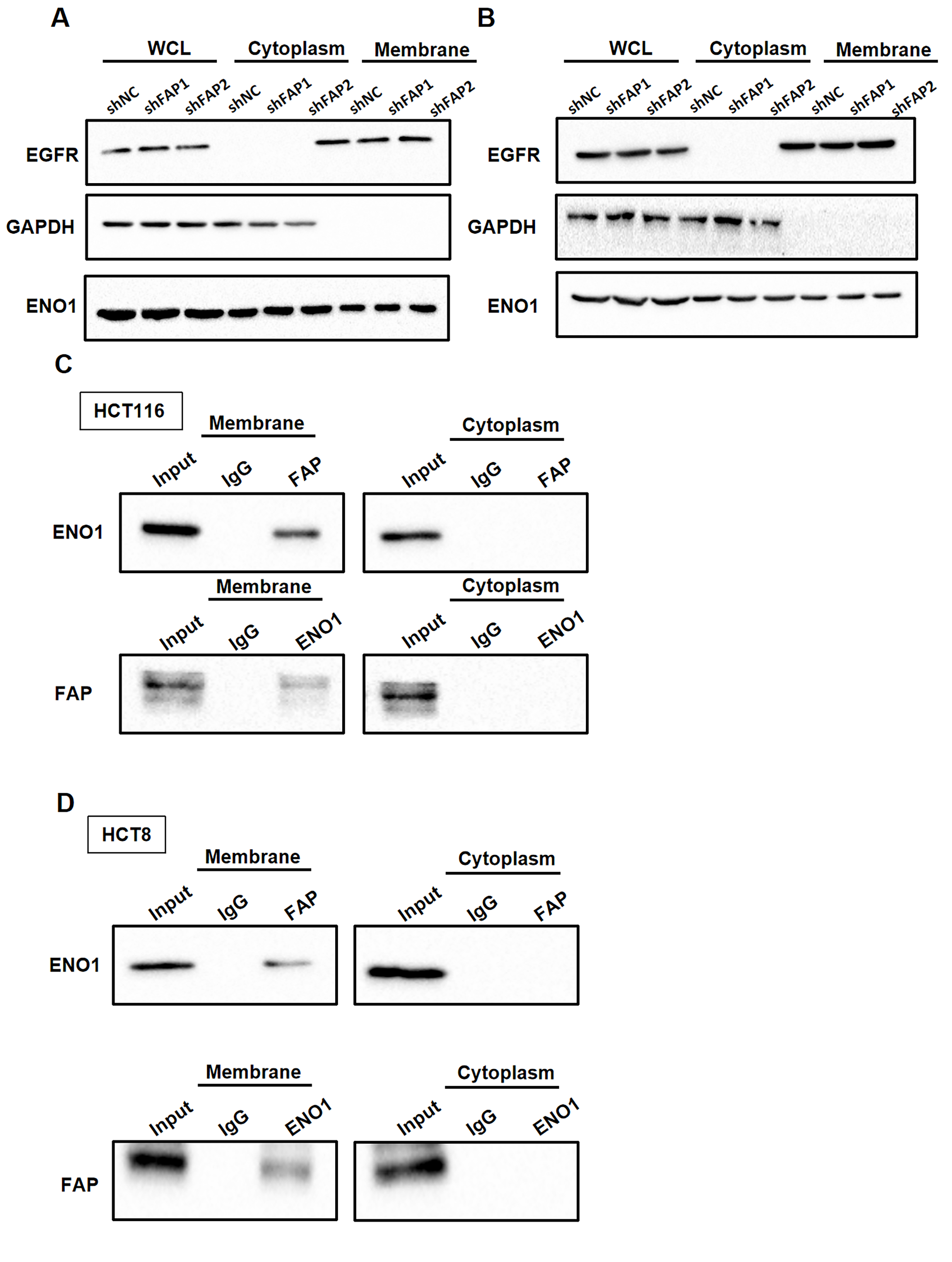

Supplement: Supplementary file 9 — supplemental figure 8 [file 41419_2021_3823_MOESM9_ESM.tif]
