# Supplementary material for: Colorectal cancer cell intrinsic fibroblast activation protein alpha binds to Enolase1 and activates NF-κB pathway to promote metastasis
Source: Cell Death Dis. 2021 May 25;12(6):543. doi: 10.1038/s41419-021-03823-4 (PMC8149633; doi:10.1038/s41419-021-03823-4)
Supplement: Supplementary file 10 — supplemental file 1 [file 41419_2021_3823_MOESM10_ESM.pdf]

RELA

NM_021975(S536D)

ATGGACGAACTGTTCCCCCTCATCTTCCCGGCAGAGCCAGCCCAGGCCTCTGGCCCCTATGTGG
AGATCATTGAGCAGCCCAAGCAGCGGGGCATGCGCTTCCGCTACAAGTGCGAGGGGCGCTCC
GCGGGCAGCATCCCAGGCGAGAGGAGCACAGATACCACCAAGACCCACCCCACCATCAAGAT
CAATGGCTACACAGGACCAGGGACAGTGCGCATCTCCCTGGTCACCAAGGACCCTCCTCACCG
GCCTCACCCCCACGAGCTTGTAGGAAAGGACTGCCGGGATGGCTTCTATGAGGCTGAGCTCTG
CCCGGACCGCTGCATCCACAGTTTCCAGAACCTGGGAATCCAGTGTGTGAAGAAGCGGGACCT
GGAGCAGGCTATCAGTCAGCGCATCCAGACCAACAACAACCCCTTCCAAGTTCCTATAGAAGA
GCAGCGTGGGGACTACGACCTGAATGCTGTGCGGCTCTGCTTCCAGGTGACAGTGCGGGACCC
ATCAGGCAGGCCCCTCCGCCTGCCGCCTGTCCTTTCTCATCCCATCTTTGACAATCGTGCCCCCA
ACACTGCCGAGCTCAAGATCTGCCGAGTGAACCGAAACTCTGGCAGCTGCCTCGGTGGGGATG
AGATCTTCCTACTGTGTGACAAGGTGCAGAAAGAGGACATTGAGGTGTATTTCACGGGACCAG
GCTGGGAGGCCCGAGGCTCCTTTTCGCAAGCTGATGTGCACCGACAAGTGGCCATTGTGTTCCG
GACCCCTCCCTACGCAGACCCCAGCCTGCAGGCTCCTGTGCGTGTCTCCATGCAGCTGCGGCG
GCCTTCCGACCGGGAGCTCAGTGAGCCCATGGAATTCCAGTACCTGCCAGATACAGACGATCG
TCACCGGATTGAGGAGAAACGTAAAAGGACATATGAGACCTTCAAGAGCATCATGAAGAAGAG
TCCTTTCAGCGGACCCACCGACCCCCGGCCTCCACCTCGACGCATTGCTGTGCCTTCCCGCAGC
TCAGCTTCTGTCCCCAAGCCAGCACCCCAGCCCTATCCCTTTACGTCATCCCTGAGCACCATCAA
CTATGATGAGTTTCCCACCATGGTGTTTCCTTCTGGGCAGATCAGCCAGGCCTCGGCCTTGGCC
CCGGCCCCTCCCCAAGTCCTGCCCCAGGCTCCAGCCCCTGCCCCTGCTCCAGCCATGGTATCAG
CTCTGGCCCAGGCCCCAGCCCCTGTCCCAGTCCTAGCCCCAGGCCCTCCTCAGGCTGTGGCCC
CACCTGCCCCCAAGCCCACCCAGGCTGGGGAAGGAACGCTGTCAGAGGCCCTGCTGCAGCTG
CAGTTTGATGATGAAGACCTGGGGGCCTTGCTTGGCAACAGCACAGACCCAGCTGTGTTCACA
GACCTGGCATCCGTCGACAACTCCGAGTTTCAGCAGCTGCTGAACCAGGGCATACCTGTGGCC
CCCCACACAACTGAGCCCATGCTGATGGAGTACCCTGAGGCTATAACTCGCCTAGTGACAGGG
GCCCAGAGGCCCCCCGACCCAGCTCCTGCTCCACTGGGGGCCCCGGGGCTCCCCAATGGCCTC
CTTTCAGGAGATGAAGACTTCTCC**GAC**ATTGCGGACATGGACTTCTCAGCCCTGCTGAGTCAGA
TCAGCTCCTAA
